# The development of a minimum dataset for MRI reporting of anorectal fistula: a multi-disciplinary, expert consensus process

**DOI:** 10.1007/s00330-022-08931-z

**Published:** 2022-06-23

**Authors:** Nusrat Iqbal, Charlene Sackitey, Arun Gupta, Damian Tolan, Andrew Plumb, Edmund Godfrey, Catherine Grierson, Andrew Williams, Steven Brown, Charles Maxwell-Armstrong, Iain Anderson, Christian Selinger, Alan Lobo, Ailsa Hart, Phil Tozer, Phillip Lung

**Affiliations:** 1 Robin Phillips’ Fistula Research Unit, St Mark’s Hospital, London, UK; 2 St Mark’s Hospital, London, UK; 3 St James’s University Hospital, Leeds, UK; 4 University College London Hospitals, London, UK; 5 Cambridge University Hospitals NHS Trust, Cambridge, UK; 6 University Hospital Southampton, Southampton, UK; 7 Guy’s and St Thomas’ Hospital NHS Foundation Trust, London, UK; 8 Sheffield Teaching Hospitals NHS Foundation Trust, Sheffield, UK; 9 Nottingham University Hospitals NHS Trust, Nottingham, UK; 10 Salford Royal Hospital, Manchester, UK

**Keywords:** Anal fistula, MRI, MRI reporting

## Abstract

**Abstract:**

There are a range of sphincter-preserving procedures available to treat anorectal fistula, some of which can be precluded, or rendered more optimal by specific features of fistula anatomy. Magnetic resonance imaging (MRI) is the gold standard modality for assessing anorectal fistula. To maximise clinical utility, the MRI report should accurately describe these clinically relevant features. We aimed to develop a minimum dataset for reporting MRI of anorectal fistula, in order to improve the assessment and management of these patients. A longlist of 70 potential items for the minimum dataset was generated through systematic review of the literature. This longlist was presented to radiologists, surgeons and gastroenterologists in an online survey to understand the features that shape current clinical practice. The longlist and survey results were then presented to an expert consensus panel to generate the final minimum dataset through discussion and anonymous voting. The final minimum dataset details the general characteristics, features of the internal and external openings, path of the fistula through the sphincters and any associated extensions and collections that should be described in all MRI reports for anal fistula. Additional surgical and perianal Crohn’s disease subsets were developed to indicate the features that aid decision-making for these patients, in addition to a minimum dataset for the clinical request. This study represents a multi-disciplinary approach to developing a minimum dataset for MRI reporting of anal fistula, highlighting the most important features to report that can assist in clinical decision-making.

**Key Points:**

*• This paper recommends the minimum features that should be included in all MRI reports for the assessment of anal fistula, including Parks classification, number of tracts, features of the internal and external opening, path of the tract through the sphincters, the presence and features of extensions and collections.*

*• Additional features that aid decision-making for surgery or in the presence of Crohn’s disease have been identified.*

*• The items that should be included when requesting an MRI are specified.*

**Supplementary Information:**

The online version contains supplementary material available at 10.1007/s00330-022-08931-z.

## Introduction

Magnetic resonance imaging (MRI) is the gold standard imaging modality for anal fistula [1, 2]. Details of fistula morphology are communicated through the radiology report which should be unambiguous, relevant and concise without placing unnecessary burden upon reporting radiologists. This is a challenge, as anal fistulae can be complex and reporting requires precise description of the spatial configuration of key features. Both free-text and structured reporting [3–5] have advantages and disadvantages, whilst a minimum dataset, set of features that should be reported for all anal fistulae, can be used in either style emphasising items that are most valuable.

Successful treatment of anal fistula relies on accurate assessment of fistula anatomy, particularly in complex cases. Surgery guided by clinical examination alone has a higher risk of recurrence due to undetected tracts, with significantly improved outcomes when MRI is utilised. Studies have shown that fistula recurrence rate is 13% when surgery is guided by MRI findings compared to 52% when there is discordance between imaging and clinical examination [3]. Imaging has further benefits in supporting surgical decision-making for the use of sphincter-preserving procedures (SPPs). These minimally invasive techniques have variable efficacy and are usually favoured where fistulotomy (requiring variable division of the sphincter muscles) is likely to result in continence disturbance. The feasibility of many SPPs is partly determined by specific anatomical features, such as tract tortuosity, diameter and the presence of intersphincteric complexity [4].

To improve clinical utility of reporting and provide a guide to radiologists when reporting fistulas, we aimed to develop a minimum dataset for MRI reporting of anal fistula, to ensure the key anatomical features are described without losing the flexibility of reporting additional findings that have clinical relevance.

## Methods

### Systematic review and clinician survey

The minimum dataset was developed in multiple stages, including a systematic review of the literature to identify articles describing recommended features to be reported on MRI, followed by a nationwide clinician survey regarding the relevant information required for decision-making. These were used to inform an expert consensus panel to determine the final dataset, ensuring that it captured regional variation of clinical assessment and management [5]. The methodology and results for these parts of the project are reported in the Supplementary Material. Ethical committee approval was not required. The recommended MR sequences and protocols required for assessment of relevant features is beyond the scope of this project, and has been described in detail elsewhere [1, 4, 6–8].

### Expert consensus panel

Clinicians who completed the survey were asked if they wished to participate in the expert consensus panel to determine the final minimum dataset. Experts were determined to be those with a combination of at least 3 of the following criteria: [1] a minimum of 6 years in practice in a consultant position, [2] perform clinical or radiological assessment of more than 50 patients with anal fistula per year, [3] have produced more than 3 publications related to anal fistula in the last 5 years, [4] receive or review tertiary referrals for anal fistula, and for surgical participants, perform more than 3 types of procedure for anal fistula, 2 of which did not include lay open or insertion of drainage seton. From this expert pool, panel members were selected to reflect gender and geographical diversity.

The invited panel met virtually to discuss and vote on the final minimum dataset. Each feature from the longlist was presented alongside results of the clinician survey, followed by panel discussion. The panel then cast anonymous votes for whether the feature should be included in the minimum dataset, with answer options consisting of ‘Always report (even if absent)’, ‘Report if remarkable or relevant to clinical scenario’ and ‘Never Report’ (see Supplementary Materials: clinician survey methods). The consensus threshold for inclusion into the minimum dataset was determined a priori as 70% of the panel voting for either ‘always report (even if absent)’, report if remarkable or relevant, or if the vote for ‘never report’ was < 30%, in which case the feature would be included under report if remarkable or relevant.

## Results

The systematic review identified 26 publications from which a longlist of 70 potential items was derived and presented in the clinician survey (see Supplementary Material) [6, 7, 9–32]. A total of 14 experts determined the final minimum dataset, including 3 gastroenterologists, 6 radiologists and 5 surgeons. The panel felt that some features, whilst being beyond the remit of a minimum dataset, were particularly relevant when planning for surgical procedures, or in the assessment of perianal Crohn’s fistula. As a result, additional voting options of ‘report within a surgical subset’ or ‘report within a perianal Crohn’s disease subset’ were introduced to indicate the specific clinical scenarios in which these features are of importance. The consensus thresholds are described in Table 1. The final minimum dataset and associated subsets are presented in Table 2, with explanatory notes in Table 3 and full results of panel voting detailed within Supplementary Tables S13–S27. Specific discussion points are detailed below.

**Table 1:** Expert consensus voting options and consensus thresholds

Voting option	Consensus threshold
Always report (even if absent*)	70% of all votes
Report if remarkable or relevant	70% of all votes or Votes for always report + remarkable/ relevant = 70%
Report in surgical subset	70% of all votes or Votes for always report + remarkable/ relevant + surgical subset = 70%
Report in pCD subset	70% of all votes or Votes for always report + remarkable/ relevant + surgical subset + pCD subset = 70%
Never report	70% of all votes

*In conditional features, such as abscesses and extensions

**Table 2:** The minimum dataset for MRI reporting of anal fistula and associated subsets

Feature	Always report	Report if remarkable or relevant to clinical scenario**
Classification	• Parks classification subtype	
General characteristics	• Number of tracts • If tract is single, single-branched or multiple	
Internal opening	• Anal clock location • Height in upper/middle/lower thirds of anal canal* • If internal opening is anal or rectal • Number of internal openings	• Diameter
Path of the fistula tract through the sphincters	• Location where tract crosses EAS or puborectalis • Height that tract crosses EAS or puborectalis in upper/middle/lower thirds of anal canal*	• General characteristics of IAS/EAS • Course of IS fistula through IS space
External opening	• Anal clock location • Anatomical location (e.g. gluteal, labial)	
Extensions	• Presence of extensions, even if absent • If extensions are single or multiple • Anatomical location • Location relative to levator ani (supra/infralevator) • Location of point of communication to primary tract • Shape (e.g. horseshoe, blind tract)	• Description of course of extensions
Collections	• Presence of collections, even if absent • Connection to the primary tract • Anal clock location • Anatomical location (e.g. perianal, ischioanal) • All collections should be reported, with size defined as [34]: • Small (3–10 mm, not including tracts > 3 mm diameter) • Medium (11–20 mm) • Large (> 20 mm) • Large collections should be notified to the referring team	• Height of collections
Measurements		• Tract length • Tract diameter
Other features	If present, comment on: • Fistula activity: fibrotic, healed or scarred tract • Rectum and large bowel: presence of proctitis, presence of small- and large-bowel inflammation • Features of previous surgery: setons, drainage catheters, air foci, gas in fistula • Other pathologies: rectal wall thickening, involvement of pelvic organs, pelvic abscess with fistulous tracts, inflammation of adjacent tissues, retrorectal cysts, bone marrow oedema, osteomyelitis, anogenital fistulation, lymphadenopathy, malignant transformation of fistula, peritoneal psuedocysts, unilateral thickening of levator ani, tuberculosis, diverticulitis • Other perianal pathology: pilonidal sinus, hydradenitis suppurativa, haemorrhoids, fissure	
Surgical subset** (report when planned surgical intervention is indicated on request)		
• Angulation through EAS/IS space • Direction through EAS (cephalad/caudad) • Angulation of branches • Distance between external opening and anal verge • Distance between extensions and primary tract • Height of extensions • Features of previous surgery: if present, comment on fat containing grafts, scarring		
Perianal Crohn’s disease subset** (report when Crohn’s disease is present or suspected on request)		
• Tract activity: active vs inactive tract		

*Length of the anal canal is defined as the length of striated muscle inclusive of puborectalis. The plane in which the canal is measured should be clearly stated

**See explanatory notes in Table 3

*EAS* external anal sphincter, *IAS* internal anal sphincter, *IS* intersphincteric

**Table 3:** Minimum dataset explanatory notes

Features to be reported if remarkable or relevant	
Internal opening diameter	• Remarkable if very large or easily visualised on MRI • Relevant in procedures where the internal opening requires closure, e.g. video-assisted anal fistula treatment (VAAFT), fistula laser closure (FiLaC). • Advancement flap: determines flap size and tension required for closure
General characteristics of IAS/EAS	• Remarkable if incomplete, thinning or poor quality, e.g. previous surgery or obstetric injury • Relevant in patients reporting incontinence, or in procedures requiring further muscle division, e.g. fistulotomy or advancement flap involving muscular layers
Course of an IS fistula through the IS space	• Remarkable if the primary tract is angulated or curving/horseshoeing • Relevant in fistulotomy, indicating the size of the wound, or if FiLaC or VAAFT is being considered, where tight angulations may preclude the procedure (Figure 5)
Description of the course of extensions	• Remarkable if the course of an extension is angulated or curving, or extends over a long distance from its origin • Relevant if the extension is to be laid open, indicating the size of the operative wound, or if VAAFT is planned, which can be precluded by tight or successive angulation of tracts
Height of collections	• Remarkable if very high—this may indicate difficulty in drainage, or may be best drained via trans-luminal route in supralevator collections • Relevant in fistulae of all aetiologies and in most surgical procedures—collections need adequate drainage to ensure the highest chances of success, and appreciating height guides the surgical procedure
Tract length	• Remarkable in very long fistula tracts • Relevant in specific surgical procedures where evidence suggests tract length is correlated with success (e.g. anal fistula plug more successful in tracts > 4 cm)
Tract diameter	• Remarkable in very wide tracts or very narrow tracts • Relevant when considering: ○ VAAFT: diameter must allow cannulation by 3.7 × 4.4 mm scope ○ FiLaC: laser penetration may be less effective in wide tracts ○ LIFT: diameter of the intersphincteric portion to be dissected and ligated ○ Plug: determines plug size
Surgical subset	
Angulation through EAS/IS space	• Fistulotomy: cephalad angulation through EAS or IS space would result in division of more muscle than expected based on assessment of internal opening alone (Figure 3) • LIFT may be precluded by angulation through the IS space as this would make dissection and ligation of the tract challenging • VAAFT and FiLaC are precluded by tight or successive angulations • Tight angulations would make plug placement more challenging
Direction through EAS (Cephalad/caudad)	• Tight or successive angulation would make procedures such as VAAFT and FiLaC more challenging • When considering fistulotomy, cephalad angulation of the tract through EAS would result in greater division of EAS than suggested by the height of the internal opening alone
Angulation of branches	• When probing the tract during examination under anaesthesia, tight or wide angulations make passage of the probe, or subsequent seton insertion more challenging, and if undetected, can raise the risk of creating a false passage • Tight or successive angulations are more difficult to negotiate with VAAFT or FiLaC (Figure 4)
Distance between external opening and anal verge	• A large distance between external opening and anal verge would result in a large wound if fistulotomy is considered
Distance between extensions and tract	• Indicating the length of extensions can identify the parts of the tract that require treatment with VAAFT, or the extent of the wound if the extension needs to be laid open
Height of extensions	• Relevant in cephalad or high extensions when a fistulotomy is being considered, as this would influence how much sphincter is to be divided
Features of previous surgery	• The presence of fat containing grafts within the fistula tract • The presence of scarring, as healing on MRI can lag behind clinical healing
Perianal Crohn’s disease subset	
• Distinction between an active or inactive tract, which can be determined by hyperintensity on T2-weighted images best seen with fat saturation for active tracts, or lack of hyperintensity on T2-weighted images for inactive tracts • Can help determine disease activity/response to treatment particularly when compared with previous scans	

### Location and height of a specific feature of fistula morphology (e.g. internal opening, branches or extensions)

The panel unanimously agreed that the anal clock descriptor should be used to describe the radial location of a feature. However, to describe a path between 2 locations, the clock face direction of travel should be specified. For example, a horseshoe tract passing clockwise from the 10 to 2 o’clock positions would describe an anterior tract, which, due to EAS being shorter in women anteriorly, would have a different management strategy to a tract passing anti-clockwise posteriorly, between the same locations.

**Fig. 1:**
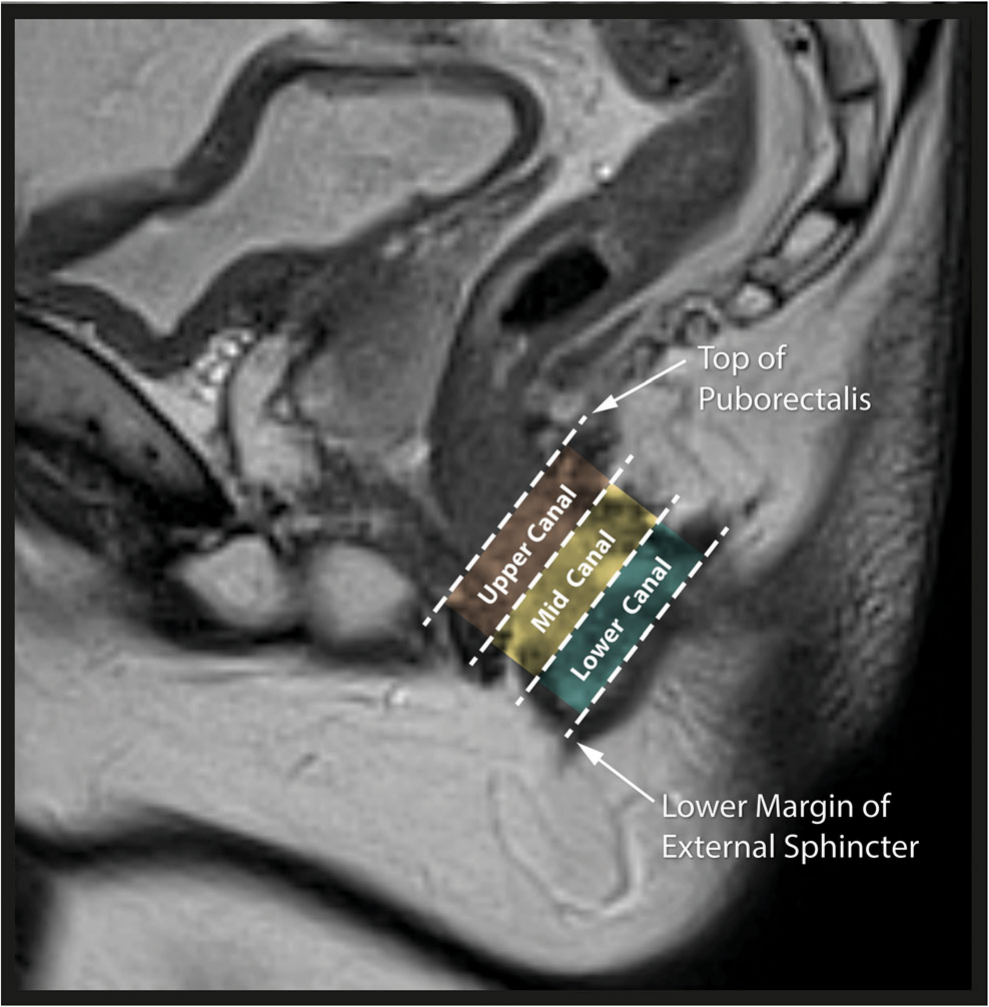
Sagittal T2 image of a 24-year-old female with Crohn’s disease: the height of a particular feature should be determined by the length of striated muscle inclusive of puborectalis and divided into upper, middle and lower thirds, as shown

The appropriate descriptor for height generated extensive discussion amongst the expert panel for several reasons:

1. There is no validated measure of height and a lack of evidence regarding superiority of any method, as well as significant inter-observer variability in the measurements obtained.
2. The lower limit of IAS is the only fixed landmark on MRI from which height can be ascertained. Distance from the anal verge frequently does not correlate with findings on clinical examination, with the patient in either left lateral or lithotomy position where there is anatomical distortion due to traction, as would be the case during Examination Under Anaesthesia (EUA). Stating height as a ratio of anal canal length (for instance in upper, middle or lower thirds) would address these issues, and require the anterior-posterior position of the fistula to be stated, given the asymmetry of anatomy.
3. Whereas a minimum dataset serves to only describe the height of a feature, in clinical practice, the height of the tract becomes synonymous with the feasibility of fistulotomy, placing additional burden on radiologists to ensure that this measurement is accurate and unambiguous. Suitability for fistulotomy is determined by assessment of clinical factors, such as bowel habit in addition to fistula anatomy [33], and should never be based on radiology alone. Nonetheless, MRI provides valuable information regarding sphincter involvement, and therefore all agreed that the minimum dataset should include some measure of height, but that this should be carefully defined.
4. The panel agreed that the reference point when determining height should be the length of striated muscle inclusive of puborectalis, and that the plane in which it is measured (either coronal or sagittal) should be clearly stated (Figure 1).

The majority (82%) of the expert panel agreed that upper, middle and lower thirds were the most appropriate descriptor, as determined by the length of striated muscle inclusive of puborectalis with the plane in which it is measured clearly stated. Furthermore, accurate assessment of fistula height is predicated on correct scan orientation, with the imaging plane aligned along the longitudinal and transverse axis of the anal canal.

### General characteristics

#### Simple vs complex

A complex fistula is defined in various ways (Table S1) [11, 20, 34]. In practice, a simple fistula could be synonymous with a fistula in which healing can be achieved, whereas a complex fistula may indicate one in which symptom control would be the appropriate goal. Although it was considered a helpful summary term, there was no consensus on whether simple/complex should be part of the minimum dataset and was therefore excluded.

### Internal opening

#### Internal opening diameter

**Fig. 2:**
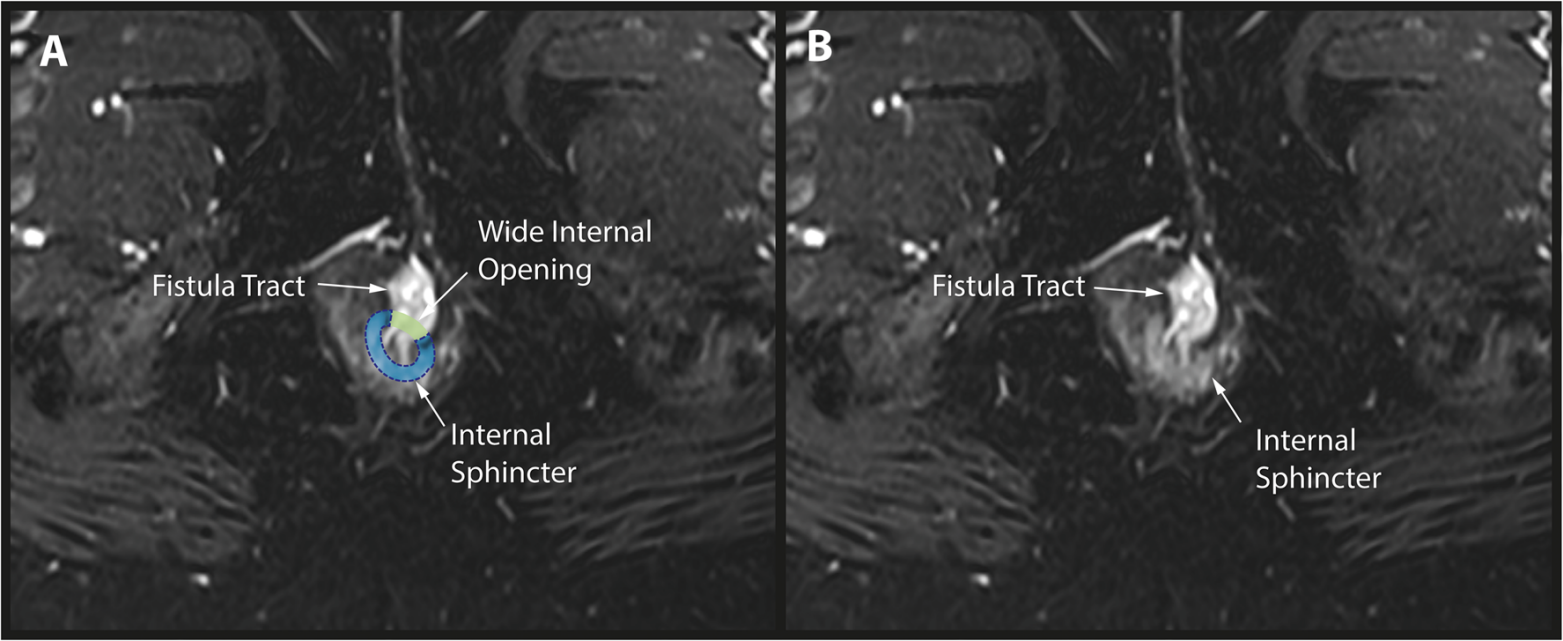
**A**, **B** Axial T2 STIR images of a 32-year-old female with an anterior fistula: the green-shaded area denotes the limits of a wide internal opening. This should be reported as it is remarkable and may impact surgical planning

Measurement of the internal opening is frequently inaccurate as the canal is collapsed and the internal opening components (sphincter defect, epithelial/granulation tissue) vary. Despite this, the importance of internal opening diameter in surgical decision-making was highlighted, particularly in specific surgical procedures (Table 3) [4]. The panel agreed that a large internal opening was deemed to be remarkable and relevant to clinical practice and therefore should be reported (Figure 2); however, due to the difficulties in taking accurate measurements, quantification of size was not considered helpful. Instead, surgeons and radiologists working together should determine the internal opening size which they consider relevant within their practice.

### Path of the fistula tract through the sphincters

**Fig. 3:**
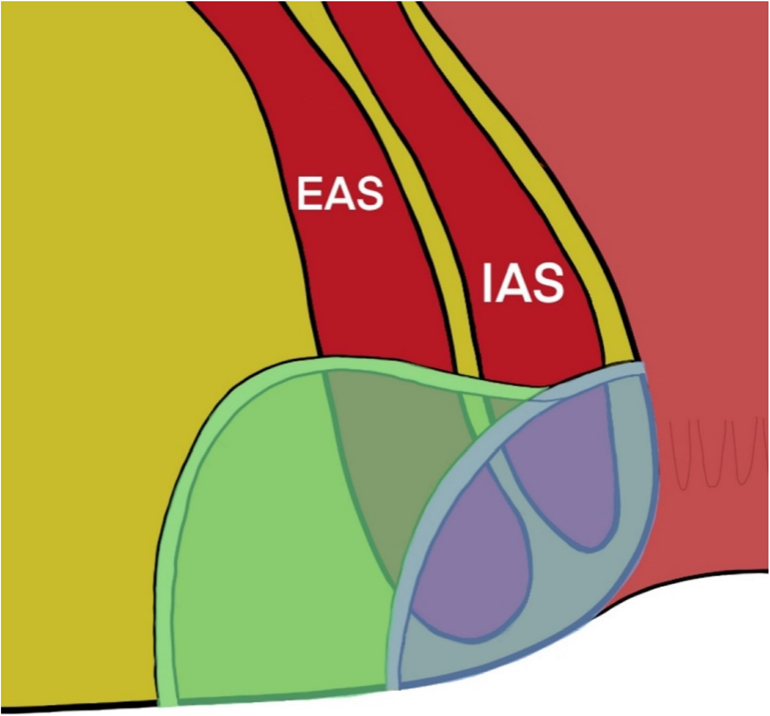
A fistula tract running obliquely cephalad from the internal opening will result in division of a greater proportion of musculature with fistulotomy. Reproduced with permission [4]

**Fig. 4:**
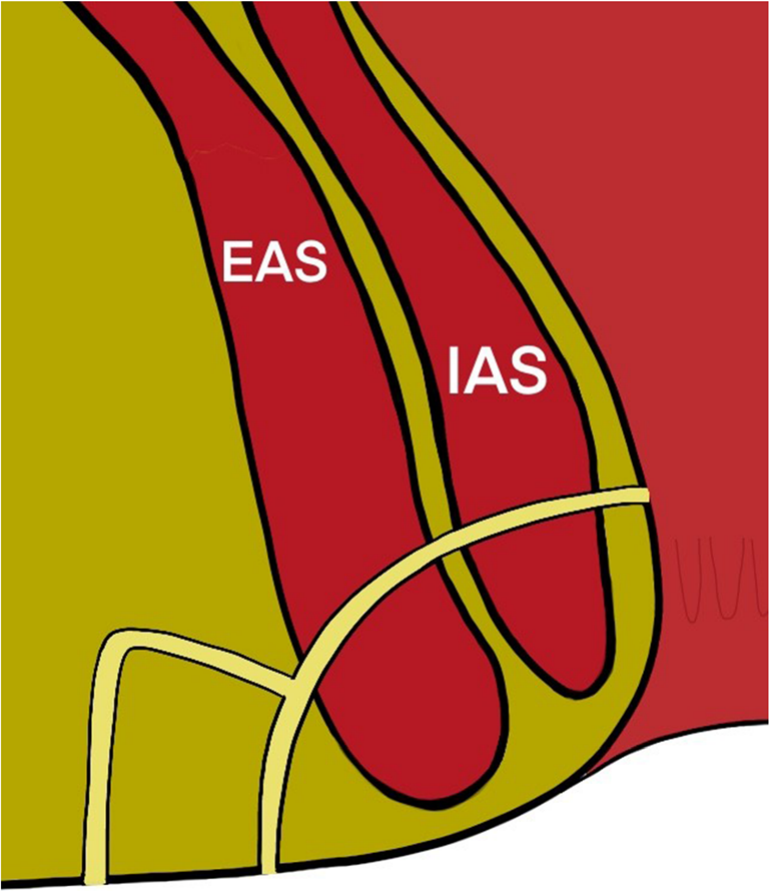
Tight or successive angulation of branches can be challenging to probe for seton insertion and to negotiate with a rigid VAAFT scope

**Fig. 5:**
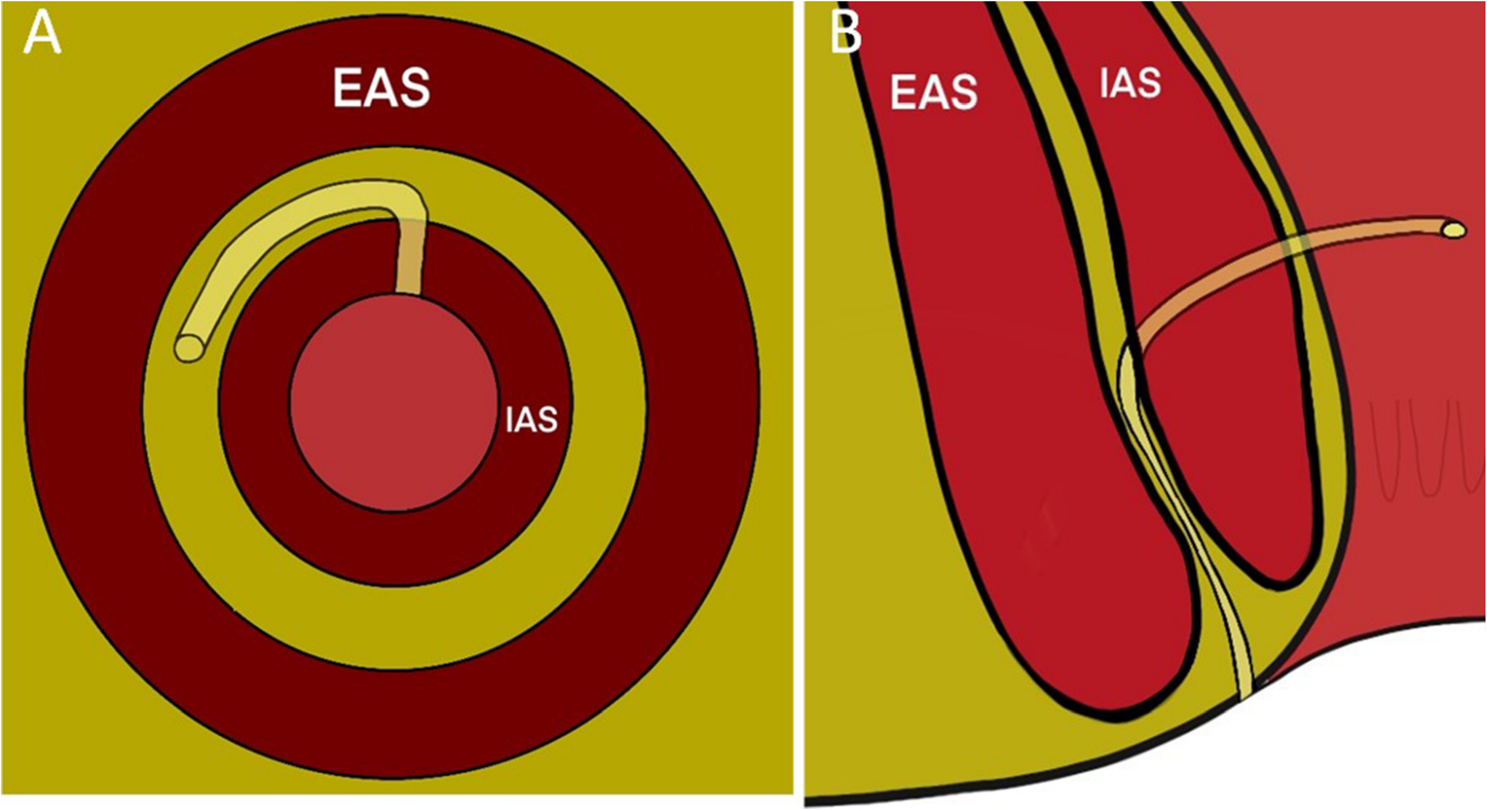
A curved or angulated intersphincteric fistula, as demonstrated here in axial (**A**) and coronal (**B**) planes, would be difficult to negotiate with a rigid fistulascope as is used in video-assisted anal fistula treatment, or using a fistula laser closure (FiLaC) probe

Details of the path of the tract through the sphincters was largely relevant to surgeons considering fistulotomy and other SPPs, as reflected in the survey results. These were therefore deemed to be important in surgical planning but beyond the scope of a minimum dataset. For example, cephalad angulation of a transphincteric fistula would result in division of a greater proportion of EAS than if the tract followed a caudad angulation (Figure 3), and tight or successive angulations make cannulation with a rigid fistulascope, as in video-assisted anal fistula treatment (VAAFT), more challenging (Figure 4). The general characteristics of internal and external anal sphincter such as length, quality and defects were deemed to be relevant to clinicians, since where the MRI indicated sphincter deficit or poor-quality musculature it may predict difficulties in maintaining continence after surgical intervention and inform clinical decision-making (Tables 2 and 3). Angulation or horseshoeing of an intersphincteric fistula through the intersphincteric space was also deemed to be remarkable or relevant as this is often indicative of a more complex morphology and is correlated with recurrence [35], as well as being an important consideration in the use of certain SPPs (Table 3, Figure 5).

### Extensions

The presence of extensions or branches is particularly relevant when considering curative surgical procedures, as untreated extensions increase the likelihood of recurrence [15, 36]. The description of the course of extensions should be described, particularly if angulated, curving or following a long course from the primary tract.

### Features of fistula activity

#### Active versus inactive tract

**Fig. 6:**
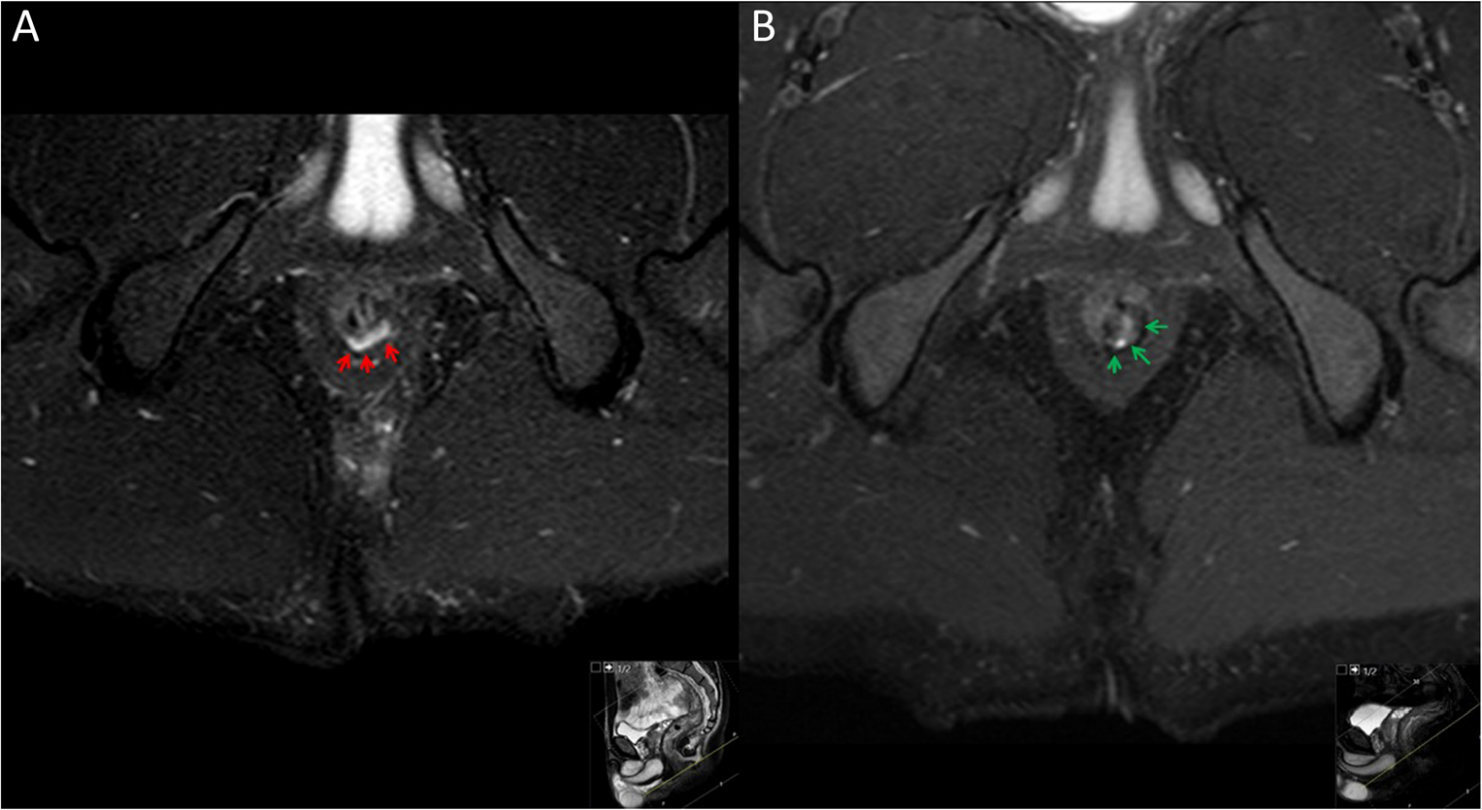
Axial T2 STIR images of a 34-year-old male with healed perianal Crohn’s disease: the image on the left (**A**) shows an active fistula tract with T2 hyperintensity, the image on the right (**B**) shows resolution becoming an inactive tract, with low signal scarring replacing the hyperintensity

The various definitions of active and inactive tracts can be seen in Table S1. The panel noted that MRI-based activity indices such as Van Assche [18] and MAGNIFI-CD [34] were based on anatomical as well as inflammatory criteria, and may be unreliable for prognostication or quantifying change over time, as well as frequently lagging behind clinical improvement [37, 38]. The inflammatory nature, as determined by features such as tissue oedema and hyperintensity on T2-weighted imaging, is useful in the follow-up of patients with Crohn’s disease once seton drainage and medical therapy have been initiated. The outcome of voting reflected that inflammatory activity of a fistula was most useful in assessment and monitoring of perianal Crohn’s disease, and therefore included in the perianal Crohn’s disease subset (Figure 6).

#### Fibrotic, healed or scarred tract

The panel noted the prognostic value of reporting a healed, fibrotic or scarred tract. Although clinical closure may occur earlier than radiological healing, the latter is a good predictor of outcome, with a longer time free from perianal events, fewer hospitalisations and perianal surgeries [39–41]. A scarred tract may have less relevance if its clinical or radiological status has remained unchanged for a prolonged period of time. The majority of the panel agreed that this should be reported if present and relevant.

#### Granulation tissue versus fluid

Differentiating granulation tissue from fluid on MRI depends on the pattern of tract enhancement with intravenous contrast. The panel noted that routine use of contrast was not universal and consequently this could not be included in the minimum dataset or subsets.

### Fistula tract measurements

The panel accepted that the measurement of fistula tract dimensions should be included where they impact decision-making. Fistula tract length has shown an association with treatment success [42] and recurrence [43, 44] in several sphincter-preserving procedures, whereas tract diameter can determine feasibility of VAAFT, where the tract has to be cannulated by a rigid 3.7 × 4.4 mm scope (Table 3).

### Other pathologies and other perianal pathologies

The alternative pathologies were identified by the systematic review as being potential causes or sequalae of anorectal fistulation, or co-existing pathologies. The panel agreed that other organs should also be searched for significant incidental pathology including endometrial and prostate malignancy.

### Collections

All specialties recognised the importance of correctly identifying collections due to the impact on continuation of biologic therapy in Crohn’s disease and to ensure adequate surgical drainage for fistulas of all aetiologies. As a result, the presence of collections, including negative reporting, is included in the minimum dataset, alongside other descriptive features such as connection to primary tract and the radial and anatomical location which help guide management.

The panel agreed that there is no clear definition for a collection on imaging, as highlighted in the European Society of Gastrointestinal and Abdominal Radiology consensus statement [1], potentially explaining contradictory free text responses in the clinician survey (summarised in Table S12). The absence of routine use of intravenous contrast across institutions may hamper differentiation between collections containing granulation tissue vs fluid, emphasising the need to correlate radiological findings with clinical assessment. However, members of the panel felt that collections should be drained regardless of content, whilst recognising that the presence of pus would affect the clinical urgency of drainage. In addition, all members agreed that size of the collection may determine further management, particularly in Crohn’s disease, and also acknowledged that clinical urgency will vary depending on how well drained they appear to be on imaging. For instance, a small, contained supralevator collection may require attention more urgently than a larger ischioanal fossa collection actively draining via the skin.

The panel agreed that all collections should be reported, and that those greater than 20 mm in diameter represent a threshold where intervention would be considered in most circumstances, and so should be highlighted to the referring team for review.

### Minimum dataset for MRI request

An effective MRI report relies on accurate and relevant information being provided by the referring clinician, and can enhance the utility of the resulting report, particularly if there is heightened awareness of important anatomical details that guide decision-making. The panel therefore agreed that a minimum dataset for MRI requests is required to generate an effective report based on a reciprocal relationship between referring and reporting teams. The minimum dataset for requests (Table 4) was generated using the same voting options and consensus thresholds described in Table 1.

**Table 4:** Minimum dataset for MRI request

Features that should be included in the MRI request	
• History of inflammatory bowel disease • Colorectal configuration (e.g. intact, ileorectal anastomosis, ileal pouch anal anastomosis) • Previous fistula surgery • Known fistula anatomy • Clinical findings and symptoms • Presence of seton • Specific clinical question Surgical subset: • State if a specific surgical procedure is planned/being proposed	

## Discussion

In our experience, there is wide variation in the quality and detail included in MRI reports for anal fistula. Reporting templates for fistula imaging have been published as recently as in the last year [45] which include features that we have described in this minimum dataset, thus affirming the relevance of the dataset and highlighting the need for a systematic approach to reporting in a way that can be easily utilised by referring clinicians. Inadequate communication across specialties can result in a mismatch between the information needed to guide clinical management and the level of detail communicated through the report. This minimum dataset aims to rectify this by emphasising multi-disciplinary discussion enabling tailored investigation and treatment to improve patient care. The minimum dataset and associated subsets can also be used as educational tools, by highlighting the clinical significance of the features described and improving the quality of reporting.

Several principles guided voting decisions throughout the consensus discussions. The primary aim was that the minimum dataset must identify the key features that should be described on all MRIs for anorectal fistula. The expert panel agreed the fundamental characteristics influencing management decisions, universal to all patients regardless of aetiology, clinical history and scope of practice of the treating centre. By definition, the minimum dataset is not exhaustive and has been constructed to allow local teams to retain flexibility to report what they know is clinically effective within their own centre. It focuses on those features that can be identified by radiologists without additional or specialist training, and we aimed to maintain simplicity to maximise uptake without additional burden on radiologists. The additional subsets were created to acknowledge that some features are of particular value in certain situations, and are highlighted to improve reporting and subsequent management of these patients. The features included in these subsets require an additional level of detail and may require more experience to interpret accurately. These are outside of the scope of what we expect to be reported as the minimum standard, but are valuable discussion points that can assist multi-disciplinary team decision-making, particularly when prompted by information in a detailed request.

Several gaps in the literature were highlighted regarding definitions, particularly where this influences subsequent management. The panel acknowledged that whilst the MRI report is not intended to dictate all aspects of management, the use of specific terminology can trigger clinical action, a prime example of which is the cessation of immunosuppressive therapies when ‘abscesses’ or ‘collections’ are identified in perianal Crohn’s disease, whereas a ‘cavity’ may alert the clinician without demanding a particular course of action, such as cessation of medical treatment. As a result, the minimum dataset avoids defining or using ambiguous terms, particularly where there is a lack of evidence supporting the definition or clinical action. Further work should be conducted into the threshold at which an EUA should occur or treatment should be paused to be able to support clinical decisions.

There are limitations to this work. Wide geographical variation in clinical practice is recognised when assessing and managing patients with anal fistula [5]. We reduced the impact of this by constructing a longlist of items for inclusion using a broad literature search with no language limitation and asked survey participants to supplement this list with missed features. No additional features were suggested or found in papers published after the literature search had been conducted, suggesting that the longlist was exhaustive. The expert panel was selected to ensure a breadth of opinion and views from individuals with expertise, a representative range of practice and geographic distribution within the UK. However, we recognise that the clinical utility of the minimum dataset is the real test and a crucial next step will be to validate it and determine its effectiveness for both radiologists and referring specialists when compared to traditional methods of reporting. Finally, a need for a minimum dataset for requesting information only became clear during the expert consensus process and was not subject to the same methodological process prior to the consensus meeting. However, there is no literature to supplement its development. Whilst a wider clinician survey may have been appropriate, the proposed ‘request’ dataset generated by the expert multi-disciplinary group is a reasonable starting point from which a more comprehensive structure can be developed in the future.

## Conclusion

This project represents the first truly multi-disciplinary endeavour to develop a minimum dataset for reporting MRI for anorectal fistula using current literature, clinical practice and expert opinion. The final dataset and surgical and perianal Crohn’s disease subsets can be used as a tool for reporting radiologists and a guide for operating surgeons to select the best treatment options for patients thus supplementing and supporting clinical practice for this challenging area of coloproctology.

## Supplementary Information

ESM 1 (DOCX 157 kb)

## Abbreviations

EAS: External anal sphincter
ESGAR: European Society of Gastrointestinal and Abdominal Radiology
EUA: Examination under anaesthesia
FiLaC: Fistula laser closure
IAS: Internal anal sphincter
LIFT: Ligation of the intersphincteric fistula tract
MRI: Magnetic resonance imaging
SPP: Sphincter-preserving procedure
VAAFT: Video-assisted anal fistula treatment

## Declaration

### Guarantor

The scientific guarantor of this publication is Dr. Phillip Lung.

### Statistics and biometry

No complex statistical methods were necessary for this paper.

### Methodology

• Systematic review

• survey

• consensus methodology

• performed at one institution
