# Supplementary material for: The development of a minimum dataset for MRI reporting of anorectal fistula: a multi-disciplinary, expert consensus process
Source: Eur Radiol. 2022 Jun 23;32(12):8306–16. doi: 10.1007/s00330-022-08931-z (PMC9705494; doi:10.1007/s00330-022-08931-z)
Supplement: Supplementary file 1 — (DOCX 157 kb) [file 330_2022_8931_MOESM1_ESM.docx]

**Supplementary material**

**Systematic review methods**

To produce a longlist of features for potential inclusion into a minimum dataset, we first conducted a review of the literature to identify the features of pelvic and fistula anatomy that should be reported in MRI, specifically in scans conducted with the aim of assessing patients with anorectal fistula of any aetiology. The protocol is registered at PROSPERO (Reference ID: CRD42019133561, available at <https://www.crd.york.ac.uk/prospero/display_record.php?RecordID=133561>) and the review is conducted in accordance with PRISMA guidelines.

Search strategy

A search strategy was devised using free-text and Medical Subject Heading (MeSH) terms for anal fistula and Magnetic Resonance Imaging. Electronic databases Medline and Embase (accessed via Ovid) were searched using the strategy detailed below, with the last search conducted on 21^st^ April 2019. There were no language limitation or methodological filters applied.

Medline

1. Anorectal fistula*
2. Rectal fistula*
3. Anal fistula*
4. Perianal fistula*
5. Fistula in ano
6. Rectal Fistula/
7. MRI
8. Magnetic resonance imaging
9. Exp Magnetic Resonance Imaging/
10. 1 or 2 or 3 or 4 or 5 or 6
11. 7 or 8 or 9
12. 10 and 11

Embase

1. Anorectal fistula*
2. Rectal fistula*
3. Anal fistula*
4. Perianal fistula*
5. Fistula in ano
6. anus Fistula/
7. MRI
8. Magnetic resonance imaging
9. Exp Magnetic Resonance Imaging/
10. 1 or 2 or 3 or 4 or 5 or 6
11. 7 or 8 or 9

Study selection

Studies were eligible for inclusion if they were peer-reviewed articles published from 2000 onwards. All article types were included if they discussed or described recommended features to be reported in MRI for anal fistula, including studies of MRI indicators of perianal disease activity and narrative reviews. Studies were excluded if they were limited to paediatric patients, or if they did not describe features to be reported in MRI for anal fistula, such as studies investigating the diagnostic accuracy of MRI.

Search results were imported and screened using Covidence software. Two reviewers (NI and CS) independently screened all titles and abstracts for eligibility, followed by full text screening and conflicts were resolved through discussion with the senior authors (PT and PL).

Data extraction

Data were extracted from eligible studies by one author (NI) using a standardised form. Study characteristics included: primary author, year of publication, type of publication and whether authorship was multidisciplinary or multi-institutional. Information regarding salient MRI features was extracted verbatim and categorised according to the part of fistula or pelvic anatomy, or general imaging characteristic to which they referred. Quality assessment was conducted using the Joanna Briggs Institute checklist for text and opinion for narrative reviews (25), or the Risk Of Bias in Non-randomised Studies- of Interventions (ROBINS-I) assessment tool (26) and the Newcastle Ottawa Scale (27) for non-randomised studies with and without a comparator respectively.

**Clinician survey: methods**

The longlist of MRI features ascertained through the literature review was refined by the senior authors (PT and PL) though an iterative process, where similar terms were combined, overlapping and duplicate items were removed and additional features that determine suitability of SPPs (4) were added if not obtained in our initial search. This final longlist was then used to develop a survey to determine the features that surgeons, radiologists and gastroenterologists found most useful in a radiology report for anal fistula.

The survey was constructed and disseminated using Qualtrics software (Qualtrics, Provo, UT, <https://www.qualtrics.com>) and opened with questions regarding participant demographics and experience. This was followed by general questions regarding the preferred fistula classification system and preferred descriptors for height and location of a particular feature. The remaining questions concerned items on the longlist. It was decided that the final minimum dataset, and therefore the response options for the survey would recognise the non-binary nature of reporting, in that some features may be valuable for diagnosis and management in specific contexts. As a result, surgeon and radiology participants were given the response options of: ‘Always report’ (with the qualifier ‘even if absent’ in features that were conditional, such as the presence of alternative pathologies); ‘Only report if remarkable/ relevant’; and ‘Never report’ for each item on the longlist. Surveys disseminated to radiologists and surgeons included free text questions for the definition of a collection, and the features that radiologists would be more likely to describe or that surgeons would like to see described if a particular SPP was planned.

Following a survey pilot, gastroenterology participants were asked about the general importance of each overall feature on a five-point Likert scale ranging from ‘Extremely important’ to ‘Not important at all’. This was based on feedback that the level of detail required for surgical assessment was less likely to influence clinical practice for gastroenterologists managing Crohn’s anal fistula.

Dissemination and survey invitations were conducted via social media and the British Society of Gastrointestinal and Abdominal Radiology online forum.

The following is an example of the survey disseminated to Surgeons & Radiologists, followed by the survey sent to gastroenterologists:

MRI Reporting for perianal fistula

Start of Block: Specialty

Dear Sir/ Madam,

We are conducting a survey to assess MRI reporting for anal fistula. The overall aim is to develop an expert consensus on the features that should be reported in all MRI for anal fistulae, which will be informed by the results of this survey.

We would be grateful if you could kindly complete the following questionnaire. All responses will remain confidential.

Please select your specialty:

- Surgeon (1)
- Radiologist (2)
- Gastroenterologist (3)

End of Block: Specialty

Start of Block: Demographics

Which town or city do you currently practice in?

________________________________________________________________

How many years have you been a Consultant Radiologist?

- 0-5 years (1)
- 6- 10 years (2)
- 11- 20 years (3)
- >20 years (4)

Do you consider yourself a:

- General Radiologist (1)
- Radiologist with a GI interest (2)
- Specialist GI radiologist (3)

What term best describes the hospital you work in?

- District General Hospital (1)
- Teaching Hospital (2)
- Tertiary referral centre (3)
- Private hospital (4)

How many MRI scans for patients with an anal fistula do you report on average, over a year?

- 0-20 (1)
- 21- 50 (2)
- 51- 100 (3)
- >100 (4)

Do you receive/ review tertiary referrals for anal fistula?

- Yes (1)
- No (2)

How many publications on anal fistula have you authored in the last 5 years?

- 0 (1)
- 1-2 (2)
- 3-5 (3)
- >5 (4)

(Surgeons only) Which of the following procedures do you perform as part of your regular practice for perianal fistula?

- Drainage seton (1)
- Cutting seton (2)
- Lay open (3)
- Advancement flap (4)
- Ligation of intersphincteric fistula tract (LIFT) (5)
- Fistula Plug (6)
- Video Assisted Anal Fistula Treatment (VAAFT) (7)
- Fistula tract Laser Closure (FiLaC) (8)
- Other (9) ________________________________________________

Would you be willing to attend an expert consensus panel to determine a standard for MRI reporting of anal fistula?

- Yes (1)
- No (2)

If you have selected yes, please enter your email address so that we can contact you with further details:

________________________________________________________________

End of Block: Demographics

Start of Block: Radiologist Survey

When reporting an anal fistula on MRI:

How would you describe the location of a particular feature of fistula anatomy (e.g. internal opening, external opening, collection)?

- Using anterior-posterior/ left-right descriptors (1)
- Using the 'anal clock' descriptor (with the patient in supine position, 12 o'clock is anterior and 6 o'clock is posterior) (2)

How would you define the height of a particular feature of fistula anatomy (e.g. the internal opening)?

- Measured in millimeters from the anal verge (1)
- Measured in millimeters from the top of puborectalis (2)
- Percentage of sphincter involved (3)
- Based on location in the upper, middle or lower third of the anal canal (4)
- Other (please specify) (5) ________________________________________________

Which classification system do you use?

- Parks classification (1)
- St James University Hospital Classification (2)
- Other (please specify) (3) ________________________________________________

When reporting an anal fistula on MRI, which of the following features should you report?

General characteristics

|  | Always report as standard (1) | Report if remarkable/ relevant to question (2) | Never report (3) |
| --- | --- | --- | --- |
| Number of tracts (1) |  |  |  |
| Single, single branched or multiple (2) |  |  |  |
| Simple or complex (3) |  |  |  |

Features of the internal opening

|  | Always report as standard (1) | Report if remarkable/ relevant to question (2) | Never report (3) |
| --- | --- | --- | --- |
| Location (as defined in Q1) (1) |  |  |  |
| Height (as defined in Q2) (2) |  |  |  |
| Whether internal opening is anal or rectal (3) |  |  |  |
| Number of internal openings (4) |  |  |  |
| Internal opening diameter (5) |  |  |  |

Features of the path of the fistula through/ above the sphincters

|  | Always report as standard (1) | Report if remarkable/ relevant to question (2) | Never report (3) |
| --- | --- | --- | --- |
| Location (as defined in Q1) where tract crosses EAS/ Puborectalis (1) |  |  |  |
| Height (as defined in Q2) that tract crosses EAS/ Puborectalis (2) |  |  |  |
| Angulation of tract through EAS/ intersphincteric space (3) |  |  |  |
| General characteristics of IAS and EAS (defects, length, quality) (4) |  |  |  |
| Course of an intersphincteric fistula through intersphincteric space (5) |  |  |  |
| Direction of the tract through EAS (e.g. cephalad, caudad etc.) (6) |  |  |  |

Features of the external opening

|  | Always report as standard (1) | Report if remarkable/ relevant to question (2) | Never report (3) |
| --- | --- | --- | --- |
| Location (as defined in Q1) (1) |  |  |  |
| Anatomical site (e.g. gluteal, labial) (2) |  |  |  |

Features of extensions

|  | Always report as standard (1) | Report if remarkable/ relevant to question (2) | Never report (3) |
| --- | --- | --- | --- |
| Presence of extensions (1) |  |  |  |
| Single or multiple (2) |  |  |  |
| Anatomical location (e.g. intersphincteric, extrasphincteric etc.) (3) |  |  |  |
| Location relative to levator ani (e.g. infra or supralevator) (4) |  |  |  |
| Location of point of communication with primary tract (5) |  |  |  |
| Shape (horseshoe, straight, blind tract) (6) |  |  |  |
| Angulation of branches (7) |  |  |  |
| Description of course of extensions (8) |  |  |  |

Features of collections

|  | Always report as standard (1) | Report if remarkable/ relevant to question (2) | Never report (3) |
| --- | --- | --- | --- |
| Presence of collections (1) |  |  |  |
| Connection to primary tract (2) |  |  |  |
| Location (as defined in Q1) (3) |  |  |  |
| Height (as defined in Q2) (4) |  |  |  |
| Anatomical location (e.g. perianal, intersphincteric, ischioanal etc.) (5) |  |  |  |

How would you define a collection?

________________________________________________________________

Features of fistula activity

|  | Always report as standard (1) | Only report if remarkable/ relevant to question (2) | Never report (3) |
| --- | --- | --- | --- |
| Active vs. inactive tract (1) |  |  |  |
| Fibrotic, healed or scarred tract (2) |  |  |  |
| Granulation tissue vs. fluid (3) |  |  |  |

Rectum and large bowel

|  | Always report as standard, **even if absent** (1) | Only report if remarkable/ relevant to question (2) | Never report (3) |
| --- | --- | --- | --- |
| Presence of proctitis (1) |  |  |  |
| Presence of small and large bowel inflammation (2) |  |  |  |

Features of previous surgery

|  | Always report as standard, **even if absent** (1) | Only report if remarkable/ relevant to question (2) | Never report (3) |
| --- | --- | --- | --- |
| Presence of seton (1) |  |  |  |
| Presence of drainage catheters (2) |  |  |  |
| Presence of fat-containing grafts (3) |  |  |  |
| Air foci- focal or low signal intensity on T1 and T2 (4) |  |  |  |
| Presence of gas in the fistula (5) |  |  |  |
| Scarring (6) |  |  |  |

Measurements regarding anal fistula

|  | Always measure as standard (1) | Only measure if remarkable/ relevant to question (2) | Never measure (3) |
| --- | --- | --- | --- |
| Tract length (1) |  |  |  |
| Tract diameter (2) |  |  |  |
| Distance between external opening and anal verge (3) |  |  |  |
| Distance between extensions and primary tract (4) |  |  |  |
| Height of extensions (as defined in Q1) (5) |  |  |  |
| Size of collections (6) |  |  |  |

Other pathologies causing fistula

|  | Always report as standard, **even if absent** (1) | Only report if remarkable/ relevant to question (2) | Never report (3) |
| --- | --- | --- | --- |
| Rectal wall thickening (1) |  |  |  |
| Involvement of pelvic organs (2) |  |  |  |
| Pelvic abscess with fistulous tracts (3) |  |  |  |
| Inflammation of adjacent tissues (4) |  |  |  |
| Retrorectal cysts (5) |  |  |  |
| Bone marrow oedema (6) |  |  |  |
| Osteomyelitis (7) |  |  |  |
| Anogenital fistulation (8) |  |  |  |
| Lymphadenopathy (9) |  |  |  |
| Malignant transformation of fistula (10) |  |  |  |
| Peritoneal psuedocysts (11) |  |  |  |
| Unilateral thickening of levator ani (12) |  |  |  |
| Tuberculosis (13) |  |  |  |
| Diverticulitis (14) |  |  |  |

Other perianal pathologies

|  | Always report as standard, **even if absent** (1) | Only report if remarkable/ relevant to question (2) | Never report (3) |
| --- | --- | --- | --- |
| Pilonidal sinus (1) |  |  |  |
| Hydradenitis Suppurativa (2) |  |  |  |
| Haemorrhoids (3) |  |  |  |
| Fissure (4) |  |  |  |

Do you perform MR fistulas with contrast?

- In every patient (1)
- Only when problem solving (please expand in box below) (2) ________________________________________________
- Never (3)

Finally, if the following named operations are planned and mentioned in the requesting information, which specific features of fistula anatomy are you more likely to describe?

- Lay open (1) ________________________________________________
- Ligation of Intersphincteric Fistula Tract (LIFT) (2) ________________________________________________
- Video Assisted Anal Fistula Treatment (VAAFT) (3) ________________________________________________
- Fistula Associated Laser Closure (FiLaC) (4) ________________________________________________
- Advancement Flap (5) ________________________________________________
- Plug (6) ________________________________________________
- Other (please specify in answer) (7) ________________________________________________

Are there any additional comments or feedback you would like to make about this survey?

________________________________________________________________

________________________________________________________________

________________________________________________________________

________________________________________________________________

________________________________________________________________

Thank you for taking the time to complete this questionnaire

End of Block: Radiologist Survey

Start of Block: Gastro survey

Which of the following features reported on MRI do you think is important to know for managing patients with anal fistula in your clinical practice as a Gastroenterologist?

|  | Extremely important (1) | Very important (2) | Moderately important (3) | Slightly important (4) | Not at all important (5) |
| --- | --- | --- | --- | --- | --- |
| General Characteristics: e.g. classification, no. of tracts, simple or complex (1) |  |  |  |  |  |
| Features of the internal opening: e.g. location, height, number, diameter (2) |  |  |  |  |  |
| Path of the fistula through the sphincters (3) |  |  |  |  |  |
| Features of the external opening: e.g. location, anatomical site (4) |  |  |  |  |  |
| Features of extensions secondary tracts: e.g. number, location, shape, height (5) |  |  |  |  |  |
| Collections or abscesses (6) |  |  |  |  |  |
| Fistula activity: e.g. active, fibrotic, healed, scarred (7) |  |  |  |  |  |
| Features of the rectum and large bowel: e.g. proctitis, inflammation (8) |  |  |  |  |  |
| Features of previous surgery: e.g. setons, scarring, air in the tract (9) |  |  |  |  |  |
| The presence of other pathologies causing fistula: e.g. pelvic sepsis with fistulous tracts, malignancy, osteomyelitis, TB (10) |  |  |  |  |  |
| Other perianal pathology: e.g. pilonidal sinus, hydradenitis (11) |  |  |  |  |  |

Are there any additional comments or feedback you would like to make about this survey?

________________________________________________________________

________________________________________________________________

________________________________________________________________

________________________________________________________________

________________________________________________________________

Thank you for taking the time to complete this survey.

End of Block: Gastro survey

**Supplemental material: Results**

Systematic Review

There were 26 publications that met the inclusion criteria (Supplemental figure S1). The majority of papers were narrative reviews based on author opinion (6–24)(Table S1) reflecting the paucity of evidence regarding the utility of anatomical features in MRI reporting. The quality of these publications was assessed using the Joanna Briggs Institute checklist for text and opinion, following which all articles were deemed to possess sufficient quality for inclusion in the study (25). The remaining studies were assessed using the Risk Of Bias in Non-randomised Studies- of Interventions (ROBINS-I) assessment tool (26) and the Newcastle Ottawa Scale (27) and are displayed in Table S1. Van Assche et al. specified features that contributed towards an MRI based score of perianal Crohn’s Disease (pCD) severity based on a prospective study of patients undergoing MRI evaluation before and after anti-TNF treatment (28). This was further refined in a modified index (29). Three studies specifically described the use of structured MRI reporting templates, developed through review of literature and collaboration between surgeons and radiologists within their respective institutions (30–32). One study used retrospective review of imaging of patients with anorectal fistula to demonstrate common MRI findings and how they should be reported (33).

The recommended features to be reported on MRI for anal fistula, as described by these papers are outlined in Table S1. These items formed the basis of the survey.

Two further papers were published in the interval between data extraction and survey closure, including one describing a minimum dataset based on expert opinion of Radiologists alone (1) and another describing an MRI based activity score for perianal Crohn’s fistula (34) (Table S2). Features identified in these publications were already included in the resulting longlist, suggesting that the initial search was exhaustive and reached saturation. However, these papers provided additional definitions of terms which were referred to in the expert consensus process.

Figure S1: PRISMA flow chart of study selection

**Identification of studies via databases**

Records removed *before screening*:

Duplicate records removed (n = 416 )

Records identified from:

Databases (n = 1641)

**Identification**

Records screened

(n = 1225)

Records excluded

(n = 1173)

Reports sought for retrieval

(n = 52)

Reports not retrieved

(n = 8, 6=abstract only, 2= unavailable)

**Screening**

Reports assessed for eligibility

(n = 44)

Reports excluded:

Do not describe MRI features (n = 16)

Duplicates (n = 1)

Paediatric population (n = 1)

Studies included in review

(n = 26)

**Included**

Table S1: Overview of studies and data extracted from literature review

| Author  Type of paper  Quality assessment | Department/  Institution | General characteristics | Internal opening | Path through sphincters | External opening | Tract measurements | Complex features | Rectum | Hyperintensity and enhancement | Previous surgery | Alternative diagnosis/ other features |
| --- | --- | --- | --- | --- | --- | --- | --- | --- | --- | --- | --- |
| Baz 2016  Retrospective review  Quality Assessment: Include^1^ | Single | SJUH | Location: anterior- posterior  Left- right  Or ‘anal clock’ | Pus in IS space |  |  |  |  | Active fistula: hypointense on T1  Hyperintense on T2  Enhances with IV contrast  Inactive: hypointense on T1, lack of T2 hyperintensity and contract enhancement |  |  |
| Chan 2015  Pictorial essay  Quality Assessment: Include^1^ | Single | SJUH-describe tract in relation to grading | Location: anal clock  Level: lower/ mid/ upper thirds of anal canal |  |  |  | Secondary tracts- course relative to sphincters and LA  Horseshoe extensions  Involvement of LA- scrutinise for pelvic pathology  Abscess- T2 hyperintense rim enhancement after Gd |  | Active: hypointense in T1, hyperintense in T2 with fat saturation  Contrast enhancement with Gd  Inactive: T1 & T2 hypointense  No contrast enhancement | Seton: linear low signal intensity structure on T1 and T2 |  |
| Cuenod 2003  Review  Quality Assessment: Include^1^ | Multi-disciplinary, multi-institution |  | High intensity area in direct contact with lumen |  |  |  | Collections: T2 hyperintense areas, air as low intensity  T1 with Gadolinium- high intensity inflammatory tissue |  | Fistula: T2 enhancement surrounded by peripheral T2 defect | Seton- pinpoint low signal intensity |  |
| deMiguel Criado 2012  Review  Quality Assessment: Include^1^ | Single | Point of origin and direction of tract using anal clock  Parks classification  SJUH |  |  |  |  | Extensions (when using Parks)  Abscesses and location (when using Parks)  Horseshoe (when using Parks)  Collections:  Contrast enhanced fat supressed T1 weighted images- central low intensity with intense ring enhancement |  | High signal intensity on T2 images  T2: hyperintense fluid in track, hypointense fibrous wall.  Active tracks- high intensity, sphincters low intensity |  |  |
| Dwarkasing 2005  Review  Quality Assessment: Include^1^ | Single | Distinction between IS and TS | Location with respect to AV and sphincter |  |  |  | Presence, size and location of secondary tracts and abscesses  Horseshoe- distinguish from TS fistula  Anovaginal fistula |  |  |  | Non-active, fibrotic tissue  Pelvic abscess with fistulous tracts  Mucinous adenocarcinoma |
| Erden 2018  Review  Quality Assessment: Include^1^ | Single | Level of canal where tract originates  Position of fistula relative to LA | Visibility and anal clock posirion | Course of fistula relative to sphincter muscles  Penetration of EAS with reference to anal clock  Integrity of sphincters | Site of external opening |  | Presence of secondary branches  Horseshoe  Presence of abscess  Anovaginal fistula as differential |  |  | Seton- string like hypointense structures looping in fistula lumen, contrast enhanced images | Pilonidal sinus: absence of internal opening or IS sepsis  Hydradenitis Suppurativa: thickening and scarring of skin, cellulitis, sinus formation, abscess and fistula in gluteal, perineal and subcutaneous tissues  Fissure: hyperintense ovoid structure on T2 |
| Gallego 2018  Review  Quality Assessment: Include^1^ | Dual specialty, single institution |  | Identify, usually at the dentate line | Identify each path and follow through entire course.  Identify communications |  |  | Abscesses and blind paths |  |  |  | Ancillary findings- inflammation of other tissues, cancer etc.  Pilonidal sinus  Haemorrhoids  Hydradenitis Suppurativa |
| George 2010  Pictorial essay  Quality Assessment: Include^1^ | Single | Identify primary track and orientation in reference to anal clock  Relation to sphincter complex- SJUH  Communication between multiple tracks | Radial clock position |  |  |  | Secondary extensions and Abscess location  Horseshoe |  |  | Hyperintensity in fat containing grafts  Seton: hypointense in hyperintense tract |  |
| Guniganti 2016  Pictorial essay  Quality Assessment: Include^1^ | Single specialty, multi-institution | Parks or SJUH  Fluid or air containing tissue, or collapsed/ granulation tissue | Clock position  Distance to skin surface on coronal images |  |  |  | Secondary fistulous tracts  Abscesses  Anovaginal fistula- presence and characterisation |  |  |  |  |
| Halligan 2003  Review  Quality Assessment: Include^1^ | Single | Fistula location- clock face  Parks classification | Location- clock face |  | Site of external opening |  | Secondary tracts and extensions- supra and infralevator  Horseshoe extensions  Abscesses |  |  |  | Rectal wall thickening- Crohn’s Disease or malignancy |
| Halligan 2006  Review  Quality Assessment: Include^1^ | Single | Parks classification  Tract in ischioanal fossa | Location- radial and level  Differentiate SS (anal internal opening) and ES (rectal internal opening) | Angulation of TS tracts through EAS |  |  | Extensions  Supralevator extension  Horseshoe |  | Active tracts- hyperintense on T2/ STIR- hypointense walls  Hyperintensity reflecting oedema  Granulation tissue: enhances but fluid hypointense on T1 |  |  |
| Ho 2019  Structured reporting template development  Quality Assessment: Include^1^ | Radiologists & Surgeons, multiple institutions | Parks- primary tract | Location- clock position  Position in anal canal/ rectum- upper, middle lower  Location confirmed in 2 planes |  | Location- clock position |  | Accessory tracts or extensions presented in visual diagrams  Supralevator sepsis  Presence and precise location of collections |  | Hypointense post-surgical fibrosis  Hyperintense- blood post-surgery  Fistula activity- active or fibrotic  Active- fluid filed- enhance with or without hypointense walls  Fibrotic tracks: T2 hypointense, no fluid or enhancement | Evidence of previous sphincter injury  Setons & drainage catheters- signal void  Hypointense post-surgical fibrosis |  |
| Horsthius 2004  Review  Quality Assessment: Include^1^ | Single | Identify tract and follow its course  Communication between multiple tracts  Parks classification | Location- anal clock |  |  |  | Extensions in levator ani, supralevator and perirectal space  Abscesses and fistulas above the levator plate  Abscesses and blind tracts |  | Post op: Hyperintensity in fat containing grafts, post op haemorrhage in T1 weighted images  Setons- hypointense |  | Bone marrow oedema  Cancer  Pilonidal Sinus extension to IS space |
| Hussain 2000  Review  Quality Assessment: Include^1^ | Radiologists & Surgeons, multiple institutions | Relation to components of the anal canal  IS versus TS | Localisation with respect to AV and sphincter complex |  |  |  | Secondary tracts- presence, size, exact location  Horseshoes  Abscesses- presence, size and exact location | Proctitis and thickened rectal fascia |  |  | Involvement of rectum, colon and small bowel loops  Unilateral thickening of LA- not due to abscess  Peritoneal psuedocysts  Rectal thickening- tumour, post-radiation, endometriosis |
| Jhaveri 2018  Review  Quality Assessment: Include^1^ | Radiologists, multiple institutions | Detection of fistula  Same classification that clinician uses  Simple or complex- (Complex tract defined as primary fistula with associated secondary tracts or abscesses) | Location- anal clock |  |  |  | Secondary tract and point of communication with primary tract  Abscesses  Presence of anogenital fistulation |  | Active tracts- high signal intensity  Chronic- low signal intensity |  | Anorectal strictures  Avascular necrosis of femoral head  Bowel disease  Malignant transformation |
| Kumar 2015  Review  Quality Assessment: Include^1^ | Surgeons and Radiologists, multiple institutions | Relationship with sphincter complex (Inter, trans, extra-sphincteric) | Location- anal clock |  | Location- anal clock | Vertical length of tract from AV  Length of tract from AV to mucosal defect | Horseshoe  Secondary tracts- single, multiple, interconnected  Supralevator extension- primary or secondary tract  Abscess- location, single vs multiple.  Widening of tract >10mm with peripheral rim/ marginal enhancement |  |  | Fat packing  Seton threads  Air foci | Ancillary findings- root cause of problem. TB, IBD, Diverticulitis, pelvic malignancy, irradiation |
| O’Malley 2012  Review  Quality Assessment: Include^1^ |  |  | Location: Anterior-posterior or anal clock | Integrity of LA- suprasphincteric or translevator disease |  |  | Secondary tracts relative to sphincters, LA and skin  Pelvic source  Abscess (See enhancement) |  | Abscess: central hyperintense signal on T2,  peripheral rim enhancement  Active tract: hypointense on T1  Hyperintense on T2 with fat saturation, enhances with IV contrast  Inactive- hypointense on T1 and T2  Lack of T2 weighted hyperintensity preceding lack of enhancement in response to treatment | Fat packing (hyperintense on T1)  Surgical drains and setons (linear low signal on T1 and T2)  Gas foci (focal low signal intensity on T1 and T2) |  |
| Sahni 2009  Review  Quality Assessment: Include^1^ | Single | Parks  Relationship of primary tract to sphincter complex | Height and radial location (clock face) |  | Location |  | Secondary tracts  Horseshoe |  | Active- high signal on T2  Fibrosed- low signal on all sequences |  |  |
| Samaan 2017  Development of MRI Index for fistulising perianal Crohn’s disease  Quality Assessment: 3^3^ | Gastroenterologists and Radiologists, multiple institutions | Single tract, single branched or multiple tracts (defined as complex tracts)  Parks |  |  |  |  | Extensions- Absent, infralevatoric, horseshow supralevatoric  Inflammatory mass- Absent, diffuce, focal. Collection: small, medium, large  Presence of recto/anovaginal tract | Rectal wall involvement- normal, thickened, increased signal intensity | Hyperintensity on T2 weighted images: absent, mild, pronounced  Hyperintensity of primary tract or extensions on post contrast T1 weighted images- absent ,mild, pronounced  Dominant feature of primary tract and extensions- fibrous, granulation tissue or fluid filled |  |  |
| Sheedy 2017  Review  Quality Assessment: Include^1^ | Single | SJUH  Parks  Van Assche score | Location: Anterior- posterior, left- right, anal clock |  |  |  | Secondary tracts- presence, course and point of communication with primary tract  Blind ending sinuses  Horseshoeing  Supralevator/ post anal abscess  Abscess- ring enhancing fluid collection of any size  Anogenital fistulation in CD |  | Hyperintense on T2, enhance with contrast  Inactive, healing or chronic- non enhancing, low intensity T1 and T2 signal intensity fibrous tracts  Diffuse homogenous enhancement of granulation tissue filled tracts |  |  |
| Szurowska 2007  Review  Quality Assessment: Include^1^ | Radiologists and gastroenterologists, single institution | Parks  State of sphincters and other pelvic organs | Location- anterior-posterior or anal clock | Passage through ischioanal/ ischiorectal fossa  Passage through EAS  Passage through LA | Location- anterior-posterior or anal clock |  | Abscess location: perianal, ischiorectal, IS, supralevator  Sepsis originating in the pelvis and tracking down through supralevator space and LA |  | Fistula activity- draining or local inflammation- intensity on T2.  Inactive- hypointense  Gas within the tract |  | Bone marrow oedema  Lymph nodes >10mm- lymphoedema/ cancer  Cancer, lymphadenopathy |
| Thippavong 2019  Review  Quality Assessment: Include^1^ | Radiologists and surgeons, multiple institutions | Parks | IO distance from AV and clock position |  | Exit site- gluteal, scrotum, vagina, labia, blind ending | Tract diameter >3mm | Secondary tracts- none, single unbranched, single branched, multiple  Extensions- Supra/ infra levator  Abscess >1cm  Anovaginal fistula | Rectal wall thickening | Hyperintensity on T2: absent/ mild/ pronounced  Enhancement within tract:  No (fluid filled)  Yes + high T2 enhancement= granulation  Yes + progressive and dark T2= fibrosis |  |  |
| Tolan 2016  Review  Quality Assessment: Include^1^ | Single | Single/ multiple tracts  Parks | Height and clock face position | Path of fistula: clock face where it enters and descends IS space (IS)  Or where it crosses EAS (TS)  Height that fistula traverses EAS | Clock position and distance from AV |  | Position of extensions: Within IS plane, Supralevator extension, Ischioanal fossa. Height and clock face position.  Horse shoeing: clock face position  Collections: position and diameter  Anogenital fistulation | Rectal wall thickening |  |  | Bone oedema and osteomyelitis  Alternative diagnoses  Malignancy in chronic fistulas |
| Torkzad 2010  Review  Quality Assessment: Include^1^ | Radiologist & Surgeon | Parks  SJUH |  |  |  |  | Secondary tracts- supralevator/ ischioanal extensions  Horseshoe  Supra/ infralevator extensions  Abscess- widening of tract >1cm | Proctitis and thickened perirectal fascia |  |  | Involvement of pelvic organs |
| Tuncyurek 2019  Development of structured reporting template- rated for clarity, helpfulness in surgical planning and completion  Quality Assessment: Moderate risk of bias^2^ | Single institution, multiple disciplines | Parks | Number  Location (Anterior-posterior, left-right and clock face)  Distance between opening and AV |  | Exit site- gluteal, scrotal, labial, vaginal, urethral (note whether left or right sided)  Note sinus tracts | Maximum tract diameter | Presence of secondary branches- None, one, multiple, describe where branch extends  Abscess: yes/ no, connected to fistula, location, size  Anovaginal fistula | Rectal/ sigmoid wall inflammation | Hyperintensity on T2:  absent/ mild/ pronounced  No central enhancement (fluid filled)  Central enhancement with high signal intensity on T2 (granulation)  Min progressive enhancement low signal intensity on T2 (fibrosis) | Setons/ drains/ prior surgeries |  |
| Van Assche 2018  Development of MRI based score of pCD severity  Quality Assessment: 5^3^ | Multi-disciplinary, single institution | Parks  Single, single branched, mulitple |  |  |  |  | Extension- supra/ infra levator  Presence or absence of abscess- Cystic lesion with clear hyperintensity on T2-weighted images and diameter >3 mm | Thickening of rectal wall | Hyperintensity on T2 weighted images (Absent/ mild/ pronounced) |  |  |

SJUH: St James University Hospital classification IS: Intersphincteric TS: Trans-sphincteric SS: Suprasphincteris ES: Extra-sphincteric Gd- Gadolinium AV- anal verge LA- Levator Ani EAS- External Anal Sphincter

1. Assessed using Joanna Briggs Institute Critical Appraisal Checklist for Text and Opinion, 2) Assessed using Risk Of Bias In Non-randomised Studies- of Interventions (ROBINS-I) assessment tool, 3) Assessed using the Newcastle Ottawa Scale

Table S2: Relevant papers outside the date range of the systematic review

| Author  Type of paper | Department/  Institution | General characteristics | Internal opening | Path through sphincters | External opening | Tract measurements | Complex features | Rectum | Hyperintensity and enhancement | Previous surgery | Alternative diagnosis/ other features |
| --- | --- | --- | --- | --- | --- | --- | --- | --- | --- | --- | --- |
| Hindryckx 2019  Development of MRI index for pCD | Multi-disciplinary, multi-institution | No. of tracts: single unbranched, complex (defined as either a single internal opening leading to >1 fistula tracts or multiple internal openings)  Location: Parks | Number: 0, 1, 2, >2 |  | Number: 0, 1, 2, 3, >3 | Length of fistula tract: <2.5cm, 2.5-5cm, >5cm | Extension: absent, infralevatoric, horseshoe configuration, supralevatoric  Inflammatory mass: Absent, diffuse, focal, small collection, medium collection, large collection  Dominant feature of primary tract and extensions: Predominantly fibrous, predominantly filled with granulation tissue, predominantly filled with fluid or pus | Presence of proctitis: absent, present | Hyperintensity of primary tract or extensions on fat saturated T2 images: Absent, mild, pronounced  Hyperintensity of primary tract or extensions on post-contrast fat saturated T1 images: Absent, mild, pronounced |  |  |
| Halligan 2020  Consensus statement | Radiologists, multiple institutions | Fistula presence  Clock face/ radial location | Radial location and level  Describe shared internal openings | Internal and external sphincter integrity | Radial location |  | Extensions: presence, anatomical location, maximal cavity diameter |  | Activity if used locally | Presence of seton | Clinical details: clinical question being asked  Associated finsings eg proctitis, osteomyelitis  Comparison with prior imaging |

Survey of current clinical opinion and practice

The survey questions are presented in Supplemental methods. A total of 118 responses were received, with 87 participants completing the survey, giving a completion rate of 74%. Complete responses were received from 28 surgeons (32%), 38 radiologists (44%) and 21 gastroenterologists (24%), with 48% of participants having more than 11 years of experience within their field and another 48% treating or scanning more than 50 patients with anal fistula per annum. Participant characteristics are displayed in Table S3. Surgeon participants were experienced in a range of procedures as demonstrated in Supplemental figure S2. Participant responses to the longlist are displayed in supplementary tables S4-S6, and the surgeon and radiologist free text responses are in tables S7-S12.

Table S3: Survey respondent characteristics

|  | **Radiologists (n=38)** | **Surgeons (n=28)** | **Gastroenterologists (n=21)** | **Total (%)** |
| --- | --- | --- | --- | --- |
| **Years in specialty at consultant level** |  |  |  |  |
| 0-5 | 8 | 7 | 8 | 23 (26) |
| 6-10 | 10 | 8 | 4 | 22 (25) |
| 11-20 | 15 | 11 | 7 | 33 (38) |
| >20 | 5 | 2 | 2 | 9 (10) |
| **Hospital setting** |  |  |  |  |
| District General Hospital | 12 | 4 | 2 | 18 (21) |
| Tertiary referral centre | 16 | 15 | 14 | 45 (52) |
| Teaching hospital | 10 | 9 | 5 | 24 (28) |
| **Scans/ patients per year** |  |  |  |  |
| 0-20 | 1 | 8 | 4 | 13 (15) |
| 21-50 | 12 | 13 | 7 | 32 (37) |
| 51-100 | 18 | 5 | 9 | 32 (37) |
| >100 | 7 | 2 | 1 | 10 (11) |
| **Do you review tertiary referrals?** |  |  |  |  |
| Yes | 14 | 20 | 19 | 53 (61) |
| No | 24 | 8 | 2 | 34 (39) |
| **No. of fistula publications in the last 5 years** |  |  |  |  |
| 0 | 31 | 13 | 8 | 52 (60) |
| 1-2 | 4 | 7 | 10 | 21 (24) |
| 3-5 | 2 | 7 | 2 | 11 (13) |
| >5 | 1 | 1 | 1 | 3 (3) |

Figure S2: Procedures routinely performed for anal fistula by surgeon survey respondents

Table S4: Survey responses from radiologists and surgeons regarding preferred descriptors for reporting the location and height of fistula features, and the preferred classification system

|  | **Radiologists** | **Surgeons** |
| --- | --- | --- |
| **Location** | | |
| Using anterior-posterior/ left-right descriptors | 0 | 0 |
| Using the 'anal clock' descriptor | 100% | 100% |
| **Height** | | |
| Measured in millimeters from the anal verge | 29% | 14% |
| Measured in millimeters from the top of puborectalis | 0% | 7% |
| Percentage of sphincter involved | 0% | 46% |
| Based on location in the upper, middle or lower third of the anal canal | 61% | 25% |
| Other (please specify) | 11% | 7% |
| **Classification system** | | |
| Parks classification (43) | 70% | 89% |
| St James University Hospital Classification | 16% | 4% |
| Other (please specify) | 14% | 7% |

Table S5: Radiologist and Surgeon responses for features that should be reported on MRI for anal fistula

| **General Characteristics** | **Radiologists: Always report** | **Surgeons: Always report** | **Radiologists: Report if remarkable/ relevant** | **Surgeons: Report if remarkable/ relevant** | **Radiologists: Never report** | **Surgeons: Never report** |
| --- | --- | --- | --- | --- | --- | --- |
| Number of tracts | 97% | 100% | 3% | 0% | 0% | 0% |
| Single, single branched or multiple | 95% | 93% | 5% | 4% | 0% | 4% |
| Simple or complex | 86% | 100% | 14% | 0% | 0% | 0% |
| **Internal opening** | | | | | | |
| Location (as defined in Q1) | 100% | 96% | 0% | 4% | 0% | 0% |
| Height (as defined in Q2) | 95% | 96% | 5% | 4% | 0% | 0% |
| Whether internal opening is anal or rectal | 89% | 79% | 11% | 21% | 0% | 0% |
| Number of internal openings | 89% | 93% | 11% | 7% | 0% | 0% |
| Internal opening diameter | 3% | 22% | 59% | 70% | 38% | 7% |
| **Features of the path of the fistula through/ above the sphincters** | | | | | | |
| Location (as defined in Q1) where tract crosses EAS/ Puborectalis | 89% | 96% | 11% | 4% | 0% | 0% |
| Height (as defined in Q2) that tract crosses EAS/ Puborectalis | 54% | 89% | 43% | 11% | 3% | 0% |
| Angulation of tract through EAS/ intersphincteric space | 16% | 32% | 47% | 61% | 37% | 7% |
| General characteristics of IAS and EAS (defects, length, quality) | 29% | 50% | 50% | 50% | 21% | 0% |
| Course of an intersphincteric fistula through intersphincteric space | 63% | 63% | 37% | 33% | 0% | 4% |
| Direction of the tract through EAS (e.g. cephalad, caudad etc.) | 47% | 63% | 45% | 33% | 8% | 4% |
| **External opening** | | | | | | |
| Location (as defined in Q1) | 100% | 89% | 0% | 11% | 0% | 0% |
| Anatomical site (e.g. gluteal, labial) | 89% | 85% | 11% | 15% | 0% | 0% |
| **Extensions** | | | | | | |
| Presence of extensions | 95% | 96% | 5% | 4% | 0% | 0% |
| Single or multiple | 92% | 96% | 8% | 4% | 0% | 0% |
| Anatomical location (e.g. intersphincteric, extrasphincteric etc.) | 84% | 96% | 16% | 4% | 0% | 0% |
| Location relative to levator ani (e.g. infra or supralevator) | 82% | 81% | 16% | 19% | 3% | 0% |
| Location of point of communication with primary tract | 71% | 78% | 29% | 22% | 0% | 0% |
| Shape (horseshoe, straight, blind tract) | 87% | 93% | 13% | 7% | 0% | 0% |
| Angulation of branches | 11% | 27% | 68% | 65% | 21% | 8% |
| Description of course of extensions | 74% | 74% | 26% | 26% | 0% | 0% |
| **Collections** | | | | | | |
| Presence of collections | 97% | 100% | 3% | 0% | 0% | 0% |
| Connection to primary tract | 87% | 93% | 11% | 7% | 3% | 0% |
| Location (as defined in Q1) | 95% | 93% | 5% | 7% | 0% | 0% |
| Height (as defined in Q2) | 53% | 82% | 42% | 18% | 5% | 0% |
| Anatomical location (e.g. perianal, intersphincteric, ischioanal etc.) | 95% | 89% | 5% | 11% | 0% | 0% |
| **Features of fistula activity** | | | | | | |
| Active vs. inactive tract | 39% | 39% | 42% | 57% | 18% | 4% |
| Fibrotic, healed or scarred tract | 66% | 57% | 29% | 39% | 5% | 4% |
| Granulation tissue vs. fluid | 37% | 30% | 47% | 63% | 16% | 7% |
| **Rectum and Large bowel** | | | | | | |
| Presence of proctitis | 32% | 64% | 66% | 32% | 3% | 4% |
| Presence of small and large bowel inflammation | 26% | 46% | 68% | 50% | 5% | 4% |
| **Features of previous surgery** | | | | | | |
| Presence of seton | 13% | 39% | 87% | 61% | 0% | 0% |
| Presence of drainage catheters | 11% | 32% | 89% | 68% | 0% | 0% |
| Presence of fat-containing grafts | 5% | 14% | 79% | 82% | 16% | 4% |
| Air foci- focal or low signal intensity on T1 and T2 | 11% | 15% | 84% | 77% | 5% | 8% |
| Presence of gas in the fistula | 11% | 21% | 84% | 75% | 5% | 4% |
| Scarring | 24% | 25% | 76% | 71% | 0% | 4% |
| **Measurements** | | | | | | |
| Tract length | 32% | 50% | 49% | 43% | 19% | 7% |
| Tract diameter | 16% | 18% | 58% | 79% | 26% | 4% |
| Distance between external opening and anal verge | 26% | 54% | 47% | 43% | 26% | 4% |
| Distance between extensions and primary tract | 13% | 36% | 55% | 57% | 32% | 7% |
| Height of extensions (as defined in Q1) | 26% | 61% | 58% | 39% | 16% | 0% |
| Size of collections | 71% | 79% | 29% | 21% | 0% | 0% |
| **Other pathologies** | | | | | | |
| Rectal wall thickening | 26% | 36% | 74% | 64% | 0% | 0% |
| Involvement of pelvic organs | 16% | 21% | 84% | 79% | 0% | 0% |
| Pelvic abscess with fistulous tracts | 26% | 25% | 74% | 75% | 0% | 0% |
| Inflammation of adjacent tissues | 18% | 25% | 82% | 75% | 0% | 0% |
| Retrorectal cysts | 11% | 14% | 87% | 86% | 3% | 0% |
| Bone marrow oedema | 8% | 4% | 92% | 96% | 0% | 0% |
| Osteomyelitis | 8% | 14% | 92% | 86% | 0% | 0% |
| Anogenital fistulation | 8% | 14% | 92% | 86% | 0% | 0% |
| Lymphadenopathy | 24% | 11% | 74% | 89% | 3% | 0% |
| Malignant transformation of fistula | 5% | 25% | 89% | 75% | 5% | 0% |
| Peritoneal psuedocysts | 0% | 0% | 89% | 96% | 11% | 4% |
| Unilateral thickening of levator ani | 3% | 11% | 84% | 86% | 13% | 4% |
| Tuberculosis | 3% | 11% | 79% | 89% | 18% | 0% |
| Diverticulitis | 3% | 7% | 97% | 93% | 0% | 0% |
| **Other perianal pathologies** | | | | | | |
| Pilonidal sinus | 3% | 4% | 97% | 96% | 0% | 0% |
| Hydradenitis Suppurativa | 3% | 7% | 95% | 93% | 3% | 0% |
| Haemorrhoids | 0% | 0% | 84% | 89% | 16% | 11% |
| Fissure | 3% | 4% | 84% | 81% | 13% | 15% |

Table S6: Gastroenterologist survey responses regarding the importance of fistula features to be reported on MRI for anal fistula

|  | **Extremely important** | **Very important** | **Moderately important** | **Slightly important** | **Not at all important** |
| --- | --- | --- | --- | --- | --- |
| General Characteristics: e.g. classification, no. of tracts, simple or complex | 63% | 21% | 16% | 0% | 0% |
| Features of the internal opening: e.g. location, height, number, diameter | 14% | 24% | 48% | 14% | 0% |
| Path of the fistula through the sphincters | 40% | 30% | 25% | 5% | 0% |
| Features of the external opening: e.g. location, anatomical site | 19% | 33% | 33% | 14% | 0% |
| Features of extensions secondary tracts: e.g. number, location, shape, height | 25% | 45% | 25% | 5% | 0% |
| Collections or abscesses | 90% | 10% | 0% | 0% | 0% |
| Fistula activity: e.g. active, fibrotic, healed, scarred | 58% | 32% | 11% | 0% | 0% |
| Features of the rectum and large bowel: e.g. proctitis, inflammation | 38% | 29% | 29% | 5% | 0% |
| Features of previous surgery: e.g. setons, scarring, air in the tract | 5% | 50% | 35% | 10% | 0% |
| The presence of other pathologies causing fistula: e.g. pelvic sepsis with fistulous tracts, malignancy, osteomyelitis, TB | 62% | 29% | 10% | 0% | 0% |
| Other perianal pathology: e.g. pilonidal sinus, hydradenitis | 24% | 38% | 33% | 5% | 0% |

Table S7: Features to report when considering Fistulotomy

|  | Lay open | Surgeons (N=25) | Radiologists (N=20) |
| --- | --- | --- | --- |
| Sphincters | Sphincter %, involvement, amount, relationship to IAS and EAS, proportion of muscle distal, height | 20 | 8 |
|  | Muscle to be left/ height relative to ARJ, IO to ARJ, IO from PR | 3 | 1 |
|  | Angulation through sphincters | 1 |  |
|  | %IAS | 1 |  |
|  | Height relative to termination of IAS | 1 |  |
|  | State of sphincter complex/ quality of sphincter/ defects | 3 | 3 |
| Fistula | Secondary tracts/ branches | 5 | 3 |
|  | Classification | 3 | 2 |
|  | Length of fistula |  | 2 |
|  | Simple vs complex |  | 2 |
|  | Integrity of fistula |  | 1 |
|  | IS course |  | 1 |
|  | Extrasphincteric component | 1 |  |
|  | Collections | 2 | 3 |
| Openings | Height/ level of IO |  | 6 |
|  | Locations of openings | 2 | 1 |
| Anal Canal | Length of anal canal | 1 | 1 |
|  | Proctitis |  | 1 |

Table S4: Features to report when considering Ligation of the Intersphincteric Fistula Tract (LIFT)

|  | LIFT | Surgeons (N=15) | Radiologists (N=13) |
| --- | --- | --- | --- |
| Sphincters | Sphincter involvement | 2 | 1 |
|  | Height of tract/ height in IS plane/ Height (of IO) relative to ARJ | 4 | 1 |
|  | Loss of IAS | 1 | 2 |
| Fistula | No of tracts | 2 |  |
|  | Course | 3 | 2 |
|  | Width and inflammation of tract | 1 |  |
|  | Classification | 5 | 1 |
|  | Extensions | 2 | 3 |
|  | Length of fistula |  | 1 |
|  | Abscess |  | 1 |
| IS space | IS scarring, IS space characteristics | 2 |  |
|  | IS complexity, sepsis, extension | 3 | 5 |
|  | Complexity (incl horseshoe) | 1 | 3 |
| Openings | Height/ location of IO/ Size of IO | 5 | 3 |
|  | EO, distance from AV | 2 |  |
| Anal canal | Scarring |  | 1 |
|  | Proctitis |  | 1 |

Table S8: Features to report when considering Video Assisted Anal Fistula Treatment (VAAFT)

|  | VAAFT | Surgeons (N=6) | Radiologists (N=7) |
| --- | --- | --- | --- |
| Fistula | Aetiology | 1 |  |
|  | Length of fistula |  | 1 |
|  | Diameter | 3 | 4 |
|  | Anatomy of tract | 2 |  |
|  | Angulation | 1 | 2 |
|  | Cavities/ abscess/ collections | 1 | 3 |
|  | Complexity |  | 2 |
|  | Classification |  | 1 |
| (Extensions) | Height, number, details of secondary tracts | 2 | 2 |
|  | SL extension | 1 |  |
| Openings | Height/ location of IO |  | 1 |

Table S9: Features to report when considering Fistula Laser Closure (FiLaC)

|  | FiLaC | Surgeons (N=3) | Radiologists (N=7) |
| --- | --- | --- | --- |
| Fistula | Diameter | 3 | 5 |
|  | Anatomy | 1 | 2 |
|  | Angulations | 1 | 3 |
|  | Length of fistula |  | 1 |
|  | Classification |  | 1 |
|  | Complexity |  | 1 |
|  | Secondary tracts |  | 1 |
|  | Fibrosis |  | 1 |
|  | Cavities/ abscess | 1 | 2 |
| Openings | Height and location of IO |  | 1 |
| Anal canal | Length of canal |  | 1 |

Table S10: Features to report when considering an Advancement Flap

|  | Advancement Flap | Surgeons (N=16) | Radiologists (N=5) |
| --- | --- | --- | --- |
| Sphincters | Quality of sphincter complex | 1 |  |
|  | Sphincter involvement | 2 |  |
|  | State of sphincter complex | 1 | 1 |
| Fistula | Height/ course of tract | 2 |  |
|  | Classification | 2 | 1 |
|  | Relation to levator | 1 |  |
|  | Complexity |  | 1 |
|  | Length of fistula |  | 1 |
|  | Extent of ischioanal disease |  | 1 |
|  | Perianal sepsis/ collections | 5 | 2 |
|  | IS sepsis/ characteristics of IS tract | 2 |  |
|  | Secondary tracts | 5 | 1 |
| Openings | EO location | 1 |  |
|  | IO height and location, number, diameter | 9 | 4 |
| Anal canal | Health of bowel/ vagina, proctitis, inflammation | 7 | 3 |
|  | Scarring/ fibrosis | 3 |  |
|  | Past complexity/ previous surgery | 1 | 1 |

Table S11: Features to report when considering an Anal Fistula Plug

|  | Plug | Surgeons (N=8) | Radiologists (N=5) |
| --- | --- | --- | --- |
| Fistula | Length of tract | 1 | 1 |
|  | Tract diameter | 1 | 1 |
|  | Collection | 2 | 1 |
|  | Height of tract | 1 |  |
|  | Course of tract | 1 |  |
|  | Classification | 2 | 1 |
|  | Secondary tracts | 5 | 2 |
|  | Angulation |  | 1 |
|  | Complexity |  | 1 |
|  | IS tract characteristics | 1 |  |
| Openings | IO characteristics | 2 | 3 |
|  | EO location |  | 1 |
| Anal canal | Proctitis/ inflammation | 1 |  |

Table S12: How survey respondents define a collection

|  | **Radiologists** | **Surgeons** |
| --- | --- | --- |
| **Size and content** | Fluid <1cm in diameter  Focal fluid pocket/ gas  >2-3mm  >10mm/ >1cm | Fluid/ pus filled cavity  Gas and thick rim  Accumulation of fluid in circumscribed location,  Free fluid sitting in any anorectal compartments >1cm Diameter of 0.5cms Diameter greater than main tract |
| **Relation to tract** | Focal dilatation in relation to diameter of tract  Focus of sepsis that is blind ending and wider than the dominant tract  Broader than primary track, larger than tract and more focal  Body of fluid within wall  Significant focal dilatation at least 3-4 times the diameter of the tract  Expanded fluid filled section of the tract | Broad tract/ tract that has increased in diameter significantly  Greater diameter than the main tract  In close proximity to or connected to main tract  Separate area from primary tract |
| **Morphology** | Well circumscribed and walled off  With or without communication to the primary tract  Clearly focal with narrow/ no entry/ exit  Well defined  Related to sphincter- either communicated with fistula or no clear connection  Rounded or oval shape, ie. Not looking like a linear tract  Discrete  Focal cavity- blind ending or poor drainage Displacing surrounding structures |  |
| **Signal** | High T2 signal content  Rim enhancing  Discrete fluid signal entity (with enhancing walls of contrast given)  High signal focus that isn’t a fistula or sinus  With no signal on T1+c  T2/ STIR hyperintense  Central non enhancing focus | High signal on T2 imaging, STIR T2 imaging |
| **Other** |  | Clinically- raised inflammatory markers and fever |

**Supplemental Material: Expert consensus panel voting results**

Table S13: Voting results for ‘How should the location of a feature be describe don MRI for anal fistula?’

| Location Descriptor | % Vote |
| --- | --- |
| ‘Anal Clock’ descriptor | 100% |
| Left- right/ anterior-posterior descriptor | 0% |

Table S14: Voting results for ‘Which classification system should be used to describe a fistula on MRI?’

| Classification | % Vote |
| --- | --- |
| Parks | 91% |
| St James University Hospital | 0% |
| Other | 9% |

Table S15: Voting results for ‘How should the height of a feature be described on MRI for anal fistula?’

| Height descriptor | % Vote |
| --- | --- |
| Measured in mm from anal verge | 0 |
| Measured in mm from top of Puborectalis | 0 |
| Percentage of sphincter involved | 0 |
| Based on location in the upper/middle/lower third of the anal canal | 82% |
| Based on location in the upper/ lower half of the anal canal | 18% |

Table S16: Voting results for General characteristics

| Feature | Always report | Report if remarkable/relevant | Never report | Outcome |
| --- | --- | --- | --- | --- |
| Number of tracts | 100% |  |  | Always report |
| Single, single branched or multiple tracts | 100% |  |  | Always report |
| Simple or Complex tract | 33% | 17% | 50% | Excluded |

Table S17: Voting results for features of the Internal opening

| Feature | Always report | Report if Remarkable/Relevant | Surgical subset | pCD subset | Never | Outcome |
| --- | --- | --- | --- | --- | --- | --- |
| Location | 100% |  |  |  |  | Always report |
| Height | 92% |  |  |  | 8% | Always report |
| Anal or rectal | 100% |  |  |  |  | Always report |
| Number of internal openings | 92% | 8% |  |  |  | Always report |
| Diameter |  | 83% |  |  | 17% | Always report |

Should diameter be quantified: Yes= 8% No=92%

Table S18: Expert panel voting results for features of the path through sphincters

| Feature | Always report | Report if remarkable/ relevant | Surgical subset | pCD subset | Never | Outcome |
| --- | --- | --- | --- | --- | --- | --- |
| Location where tract crosses EAS/ PR | 92% | 8% |  |  |  | Always report |
| Height that tract crosses EAS/ PR | 83% | 8% | 8% |  |  | Always report |
| Angulation through EAS/ IS space | 8% | 33% | 33% |  | 25% | Surgical subset |
| General characteristics of EAS IAS | 42% | 42% | 17% |  |  | Remarkable/ relevant |
| Course of IS fistula through IS space | 67% | 17% | 17% |  |  | Remarkable/ relevant |
| Direction through EAS (cephalad/ caudad) | 17% | 42% | 25% |  | 17% | Surgical subset |

Table S19: Expert panel voting results for features of the External opening

| Feature | Always report | Report if remarkable/ relevant | Surgical subset | pCD subset | Never | Outcome |
| --- | --- | --- | --- | --- | --- | --- |
| Location (clock face) | 100% |  |  |  |  | Always report |
| Anatomical site | 92% | 8% |  |  |  | Always report |

Table S20: Expert panel voting results for features of Extensions

| Feature | Always report | Report if remarkable/ relevant | Surgical subset | pCD subset | Never | Outcome |
| --- | --- | --- | --- | --- | --- | --- |
| Presence of extensions* | 92% |  | 8% |  |  | Always report |
| Single or multiple | 100% |  |  |  |  | Always report |
| Anatomical location | 92% |  | 8% |  |  | Always report |
| Description of course of extensions | 69% | 15% | 15% |  |  | Remarkable/ relevant |
| Location relative to LA | 85% | 8% | 8% |  |  | Always report |
| Location of point of communication to primary tract | 85% | 8% | 8% |  |  | Always report |
| Shape (horseshoe, blind tract) | 85% | 15% |  |  |  | Always report |
| Angulation of branches |  | 38% | 54% |  | 8% | Surgical subset |

Table S21: Expert panel voting results for features of collections

| Feature | Always report | Report if remarkable/ relevant | Surgical subset | pCD subset | Never | Outcome |
| --- | --- | --- | --- | --- | --- | --- |
| Presence of collections* | 92% | 8% |  |  |  | Always report |
| Connection to primary tract | 100% |  |  |  |  | Always report |
| Location | 100% |  |  |  |  | Always report |
| Height | 38% | 62% |  |  |  | Remarkable/ relevant |
| Anatomical location | 92% | 8% |  |  |  | Always report |

*even if absent

Table S22: Expert panel voting results for features of fistula activity

| Feature | Always report | Report if remarkable/ relevant | Surgical subset | pCD subset | Never | Outcome |
| --- | --- | --- | --- | --- | --- | --- |
| Active vs inactive | 15% | 31% |  | 54% |  | pCD subset |
| Fibrotic, healed, scarred | 23% | 62% |  | 15% |  | Remarkable/ relevant |
| Granulation tissue vs fluid | 8% | 38% |  | 15% | 38% | Excluded |

Table S23: Expert panel voting results for features of the Rectum and large bowel

| Feature | Always report- **even if absent** | Rem/Rel | Surgical subset | pCD subset | Never | Outcome |
| --- | --- | --- | --- | --- | --- | --- |
| Presence of proctitis | 23% | 69% |  | 8% |  | Remarkable/ relevant |
| Presence of small and large bowel inflammation | 15% | 69% |  | 15% |  | Remarkable/ relevant |

Table S24: Expert panel voting results for features of previous surgery

| Feature | Always report even if absent | Rem/Rel | Surgical subset | pCD subset | Never | Outcome |
| --- | --- | --- | --- | --- | --- | --- |
| Presence of seton | 8% | 85% | 8% |  |  | Remarkable/ relevant |
| Presence of drainage catheters | 8% | 85% | 8% |  |  | Remarkable/ relevant |
| Presence of fat containing grafts |  | 69% | 15% |  | 15% | Surgical subset |
| Air foci |  | 77% |  |  | 23% | Remarkable/ relevant |
| Presence of gas in fistula |  | 77% |  |  | 23% | Remarkable/ relevant |
| Scarring |  | 69% | 8% |  | 23% | Surgical subset |

Table S25: Expert panel voting results for Measurements

| Feature | Always report | Rem/Rel | Surgical subset | pCD subset | Never | Outcome |
| --- | --- | --- | --- | --- | --- | --- |
| Tract length | 17% | 58% | 17% |  | 8% | Remarkable/ relevant |
| Tract diameter | 8% | 75% | 17% |  |  | Remarkable/ relevant |
| Distance between EO and AV | 17% | 33% | 25% |  | 25% | Surgical Subset |
| Distance between extensions and primary tract | 17% | 50% | 33% |  |  | Surgical subset |
| Height of extensions | 17% | 42% | 33% |  | 8% | Surgical subset |
| Size of collections | 50% | 50% |  |  |  | Remarkable/ relevant |

Table S26: Expert panel voting results for features of Other pathologies

| Feature | Always report | Report if remarkable/ relevant | Surgical subset | pCD subset | Never | Outcome |
| --- | --- | --- | --- | --- | --- | --- |
| Rectal Wall thickening | 8% | 92% |  |  |  | Remarkable/ relevant |
| Involvement of pelvic organs | 8% | 92% |  |  |  | Remarkable/ relevant |
| Pelvic abscess with fistulous tracts | 8% | 92% |  |  |  | Remarkable/ relevant |
| Inflammation of adjacent tissues | 8% | 92% |  |  |  | Remarkable/ relevant |
| Retrorectal cysts |  | 92% | 8% |  |  | Remarkable/ relevant |
| Bone marrow cysts |  | 100% |  |  |  | Remarkable/ relevant |
| Osteomyelitis |  | 100% |  |  |  | Remarkable/ relevant |
| Anogenital fistulation |  | 100% |  |  |  | Remarkable/ relevant |
| Lymphadenopathy |  | 100% |  |  |  | Remarkable/ relevant |
| Malignant transformation of fistula |  | 100% |  |  |  | Remarkable/ relevant |
| Peritoneal psuedocysts |  | 92% | 8% |  |  | Remarkable/ relevant |
| Unilateral thickening of levator ani |  | 85% | 8% |  | 8% | Remarkable/ relevant |
| Tuberculosis |  | 92% | 8% |  |  | Remarkable/ relevant |
| Diverticulitis |  | 100% |  |  |  | Remarkable/ relevant |

Table S27: Expert panel voting results for features of Other perianal pathology

| Feature | Always report | Report if remarkable/ relevant | Surgical subset | pCD subset | Never | Outcome |
| --- | --- | --- | --- | --- | --- | --- |
| Pilonidal sinus | 8% | 92% |  |  |  | Remarkable/ relevant |
| HS |  | 100% |  |  |  | Remarkable/ relevant |
| Haemorrhoids |  | 77% | 8% |  | 15% | Remarkable/ relevant |
| Fissure |  | 92% | 8% |  |  | Remarkable/ relevant |
